# Using the diffusion of innovations theory to understand factors associated with COVID-19 vaccination among tobacco users

**DOI:** 10.1371/journal.pone.0309780

**Published:** 2024-12-12

**Authors:** Gail Carmen D’Souza, Jessica M. Yingst, Nicolle M. Krebs, Candace Bordner, Sophia I. Allen, William A. Calo, Andrea L. Hobkirk, Jonathan Foulds

**Affiliations:** 1 Department of Public Health Sciences, Pennsylvania State University College of Medicine, Penn State University, Hershey, Pennsylvania, United States of America; 2 Penn State Cancer Institute, Hershey, Pennsylvania, United States of America; 3 Department of Psychiatry and Behavioral Health, Pennsylvania State University College of Medicine, Penn State University, Hershey, Pennsylvania, United States of America; University of Macerata: Universita degli Studi di Macerata, ITALY

## Abstract

The coronavirus disease (COVID-19) pandemic has spread in the US with more than 100 million human infections and a million deaths since March 2020. A population of concern are high-risk individuals such as adults who are tobacco users, since COVID-19 is a respiratory disease that affects the lungs. Although 79% of the US population ≥ 18 years of age have completed the primary COVID-19 vaccine series; it is imperative to understand the factors associated with receiving or declining the COVID-19 vaccine among high-risk populations to improve vaccination rates. Guided by the diffusion of innovations (DOI) theory, this study identified factors associated with COVID-19 vaccination and the impact on COVID-19 vaccine uptake in adults who use tobacco. We conducted a cross-sectional study using a sample of Pennsylvanian adult tobacco users by sending a unique survey link to 4,081 email addresses in April 2022. Participants were asked about tobacco use, COVID-19 vaccination status, and reasons for receiving/declining the COVID-19 vaccine. Participants (n = 157) were 75% female, 96% White, 74% current tobacco users, and had a mean age of 50.1 (SD = 10.8) years. Nearly 78% (n = 119) received at least one dose of the COVID-19 vaccine (primary series). We categorized all vaccinated tobacco users into adopter categories of the DOI theory; innovators (10%), early adopters (14%), early majority (33%), late majority (11%), and laggards (32%). The major reason that prompted participants to get the COVID-19 vaccine was to ensure they were well protected against COVID-19 infection (77%). Additionally, the only reason for receiving the vaccine that significantly predicted early vaccine uptake (being an innovator or early adopter) was “to loosen restrictions on mask mandates and social/physical distancing” (p = 0.0180). Among the 22% that did not receive a COVID-19 vaccine, the most common major reason they declined the vaccine was because they felt politics played a big role in the vaccine development process (94%). Our findings suggest that major f actors that influenced why adult tobacco users would receive or decline the COVID-19 vaccine included infection control mandates, protection from the COVID-19 infection, and politics. Investigating these factors can help public health professionals design or develop future vaccination programs for high-risk populations in order to scale up vaccination rates.

## Introduction

Tobacco smoking is the leading cause of preventable death and disease in the US [1]. Also, tobacco use is a known risk factor for many respiratory diseases such as chronic obstructive pulmonary disease (COPD), and lung cancer [1, 2]. In 2017, the Behavioral Risk Factor Surveillance System (BRFSS) recorded the age-adjusted prevalence of COPD among current cigarette smokers as 15.2% compared to 2.8% among adult non-smokers [2].

Similarly, recent studies concluded that people who smoke cigarettes are at a greater risk of developing severe disease from COVID-19 than non-smokers [3, 4]. Cigarette smoking weakens the immune system and negatively affects the lungs, resulting in worse health outcomes (such as infection, hospitalizations, and death) among adults who are current smokers infected with COVID-19 [3, 4]. Additionally, the association of tobacco use with comorbidities also increases the likelihood of worse COVD-19 [5].

**Fig 1:**
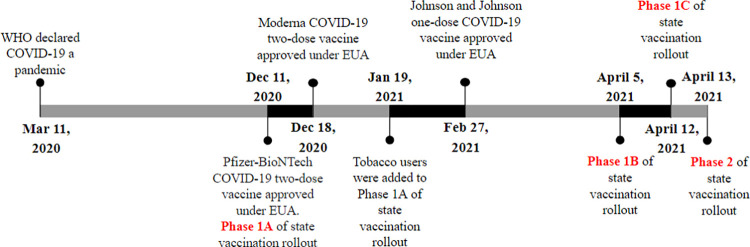
COVID-19 vaccine roll-out phases in Pennsylvania.

The 2-dose Pfizer-BioNTech (Pfizer) COVID-19 vaccine was the first to be approved by the Food and Drug Administration (FDA) under Emergency Use Authorization (EUA) on December 11, 2020 [6]. Subsequently, the Moderna (two-dose) and the Janssen (one dose) vaccines were also approved under the same conditions on December 18, 2020, and February 27, 2021, respectively [7].The COVID-19 vaccine roll-out was conducted in phases (Fig 1). For example, in Pennsylvania (PA), Phase 1a began in December 2020 and included the vaccinations of health care workers (HCWs) and long-term care facility residents, followed by smokers, who were added to Phase 1a in January 2021 [8].

A survey conducted from December 2020 to January 2021 highlighted a low COVID-19 vaccine uptake among tobacco and marijuana smokers in 2019 [9]. The COVID-19 vaccine was eligible to these users towards the end of that study, and researchers found that 49% (N = 190/387) of participants were willing to receive a COVID-19 vaccine, 26% were unwilling to get a COVID-19 vaccine, and almost 25% were “not sure” [9]. Another study conducted in PA during April 2021 (three months after tobacco users were eligible to receive the COVID-19 vaccine) highlighted that nearly 60% of participants received at least 1 dose of the COVID-19 vaccine, but among those who did not, 84% were somewhat or very unlikely to receive the vaccine [10]. Distrust of the lack of trust in the safety of the vaccine, fears about side effects, and lack of research on the efficacy and side effects of the COVID-19 vaccine may have influenced vaccine acceptance in tobacco users [10]. Also, low vaccine uptake has been prevalent in smokers prior to the pandemic, as previous evidence for the flu vaccine suggests smokers may be less likely to be vaccinated against flu as compared to non-smokers [9].

This study aimed to identify factors associated with COVID-19 vaccine uptake using the diffusion of innovations (DOI) theory. The DOI theory explains how a product or an idea gains acceptance and diffuses through a specific group or social system [11]. For this study, the DOI was used to help us understand the pattern of vaccine uptake as well as the factors associated with early uptake among tobacco users. Our study 1) used DOI to classify tobacco users into five groups (i.e., innovators, early adopters, early majority, late majority, and laggards) based on their adoption of the vaccine, 2) assessed factors associated with the uptake or decline of COVID-19 vaccination among tobacco users, and 3) identified individuals who tobacco users were most likely to trust to advise about the COVID-19 vaccine.

## Methods

### Study population

Study participants were individuals from a tobacco research registry who previously agreed to be contacted for future research at the Penn State College of Medicine in Hershey, PA. Participants were entered into the registry from August 2015 to April 2022. Email invitations with a unique survey link were sent to 4081 valid email addresses on April 28, 2022. This was approximately 1 year and 3 months after tobacco users were eligible to receive the COVID-19 vaccine in PA. Starting April 13, 2021, all Pennsylvania residents were eligible for the COVID-19 vaccine [12]. The Penn State College of Medicine Institutional Review Board approved the study protocol (Protocol #- STUDY14949).

### Measures

We used Research Electronic Data Capture (REDCap) to collect and store data from participants. REDCap is a secure, web-based application designed to support data capture for research studies [13]. For participants who did not respond to the initial invitation, four subsequent weekly reminders were sent via email. Upon accessing the unique survey link, participants were provided with the summary of research and their consent to participate were obtained by clicking continue with the survey. All participants who were at least 21 years of age and could read and write in English were included in the analysis. Participants were asked questions about their demographic information, including their age, sex, race, ethnicity, and education level. Additionally, information was gathered about their current tobacco use. Participants were asked if they used cigarettes, electronic cigarettes/vape pens, cigars, pipes, snus/snuff/dip, chew, hookah/waterpipe, or dissolvables (yes/no). Individuals that responded “yes” were then asked to indicate the type of products used, frequency of use per day, and the total number of times all products were used in a year. Dual users were categorized as participants who reported current use of both, cigarettes and e-cigarettes.

Participants were also asked about their COVID-19 vaccination status. Participants were asked if they had received at least one dose of a COVID-19 vaccine?” (yes/no). Participants who responded “yes” were then asked to indicate the type of COVID-19 vaccine they received (one dose (Johnson &Johnson), two doses (Pfizer or Moderna), don’t know). Participants that indicated receiving “one dose” or “two doses” were then asked to state which month they received their first dose or first and second dose, respectively. At the time of data collection, for surveillance purposes, the Centers for Disease Control and Prevention (CDC) defined “fully vaccinated” as “people who have received two doses on different days (regardless of time interval) of the two-dose mRNA series or received one single dose of a single-dose vaccine” [14].

To understand reasons for receiving a COVID-19 vaccine, participants who did receive the vaccine were then asked what prompted them to receive a COVID-19 vaccine. Participants had to select major reason, minor reason, or not at all, for each of the 14 reasons listed, such as, “to protect me against COVID-19 infection”, “to protect me against COVID-19 variants”, and “to return to pre-pandemic social activities” (See S1 Appendix). To understand factors related to COVID-19 vaccine hesitancy, participants who did not receive a COVID-19 vaccine were then provided with a list of reasons and asked to indicate whether each reason was a major reason, minor reason, or not a reason for why they declined/chose not to receive the COVID-19 vaccine (See S1 Appendix).

Participants were also asked to rate, “How much do you trust each of the following to advise you about the COVID-19 vaccine?” on a 4-point Likert scale of (a great deal, a good amount, not very much, not at all) for each of the 13 options, such as, “President Biden”, “Dr. Anthony Fauci”, and “Governor Wolf” (See S1 Appendix). The survey items were developed using the Kaiser Family Foundation and Washington Post survey COVID-19 Vaccine Hesitancy items.

### Applying the DOI theory to COVID-19 vaccine uptake

For the purposes of this study, we used the DOI theory to understand uptake patterns of COVID-19 vaccination and what motivated tobacco users to do so. We categorized all vaccinated tobacco users into the five adopter categories of the DOI theory: innovators, early adopters, early majority, late majority, and laggards. According to Rogers, innovators are individuals who want to try the innovation first [11]. These individuals are interested in new ideas and exploring new ventures [15]. In this study, innovators were classified as individuals who received the COVID-19 vaccine during December 2020 and January 2021. Early adopters represent opinion leaders who are comfor in adopting new ideas early in its life cycle and do not wait for many to accept the idea [11, 15]. For our study, early adopters were classified as those who received the COVID-19 vaccine during February 2021.

Early majority are rarely leaders, but they usually adopt new ideas once they have seen evidence that the innovation works [11, 15]. In our population, early majorities were classified as those who received the COVID-19 vaccine during the months of March—April 2021. These were the months when the vaccine became available to all individuals aged sixteen and above [12]. By waiting until then, tobacco users could “wait and see” the effects of the vaccine on other people, and then receive the vaccine [10]. Next, late majority were classified as people who were reluctant to change and usually adopt an innovation once it has been tried by many people [11, 15]. For this study, late majority were those who received the vaccine during the months of May-July 2021 since a majority of the public was vaccinated by then. According to the Kaiser Family Foundation Vaccine Monitor that tracked US public’s experiences with COVID-19, between May 2021 and July 2021, the number of US adults that received at least one dose of the COVID-19 vaccine increased from 62% to 67% [16, 17]. Finally, laggards were those who were conservative and extremely skeptical of change, sometimes being those who don’t ever accept the innovation [11, 15]. Among our sample, laggards were classified as those who received the vaccination from August 2021-May 2022, and unvaccinated people. People who received the vaccination from August 2021 to May 2022 may have been skeptical of the vaccine and resistant to change, or may have waited for FDA approval which was only obtained in August 2021 [7]. Unvaccinated people included those who may not have wanted to receive the vaccine for personal reasons, those who may have a history of allergic reactions to vaccines, or those who may have health reasons for being unable to receive the COVID-19 vaccine [18].

### Quantitative data analysis

Participants who were tobacco users just prior to the COVID-19 pandemic and answered the question about their COVID-19 vaccination status were included in the analysis. Descriptive statistics were presented to summarize the sample characteristics. Means and frequencies assessed reasons for why participants did or did not receive the COVID-19 vaccine. Frequency distribution was also used to classify people according to the adopter categories of the DOI theory based on the month of receiving the first dose of the COVID-19 vaccine.

A logistic regression model (Model 1, n = 136) was used to predict vaccination status (outcome = received at least 1 dose of the vaccine). Participants were excluded from the model (n = 22) if they had any missing data within the variables included in the model. Independent variables included in the model were age, sex, race, ethnicity, education, cigarette user before the pandemic, received a flu vaccine, trust in people to advise them about the COVID-19 vaccine (refer to S1 Appendix). Another model (Model 2, n = 108) was used to predict vaccinated people who were more likely to be an innovator or adopter (outcome = innovator or adopter vs. others), among those who reported being vaccinated. Participants with missing model variable data were excluded from the model (n = 15). Independent variables included in the model were age, sex, race, ethnicity, education, cigarette user, received a flu vaccine, trust in people to advise them about the COVID-19 vaccine and reasons for receiving the COVID-19 vaccine (refer to S1 Appendix). A *P*-value of less than .05 was considered statistically significant. All analyses were conducted using SAS version 9.3.

## Results

A total of 231 unique participants responded to our survey. Among them, 184 were using a tobacco product in the months immediately before the onset of the pandemic. Of the 184 participants, 157 participants answered the question about receiving one dose of the COVID-19 vaccination, while the rest (n = 27) had missing data. Complete data from 157 participants were analyzed. They were 75% female, 96% White with a mean age of 50 years (SD = 10.81). Nearly 13% earned a college degree, most (74%) reported current cigarette smoking at the time of the survey, and almost 20% reported current exclusive e-cigarette usage. Among those who reported tobacco use, around 8% were dual users (Table 1).

**Table 1:** Sample characteristics of a Pennsylvania sample of tobacco users.

	Overall (n = 157)	At least one dose (n = 122)	Not vaccinated (n = 35)
*Participant characteristics*			
Mean age in years (SD)	50.17 (10.81)	50.98 (11.24)	47.34 (8.72)
Female % (n)	74.68 (118)	72.13 (88)	85.71(30)
White % (n)	95.54 (150)	95.08 (116)	97.14 (34)
Hispanic or Latino	1.28 (2)*	0.82 (1)	2.94 (1)
Earned college degree	12.74 (20)	15.57 (19)	2.86 (1)
*Smoking characteristics*			
Cigarette smoker	73.89 (116)	75.41 (92)	68.57 (24)
Mean cigarettes per day (SD)	16.04 (8.03) (n = 116)	16.32 (8.25) (n = 92)	15.0 (7.18) (n = 24)
Mean years smoked (SD)	31.47 (11.38) (n = 127)	32.37 (11.64) (n = 102)	27.8 (9.53) (n = 25)
*E-cigarette characteristics*			
E-cigarette user	19.75 (31)	15.57 (19)	34.29 (12)
Mean times per day (SD)	12.61 (11.41) (n = 31)	11.79 (9.72) (n = 19)	13.92 (14.05) (n = 12)
Mean years of use (SD)	2.95 (2.36) (n = 30)	3.39 (2.05) (n = 19)	2.18 (2.75) (n = 11)
Other tobacco user	3.82 (6)	3.28 (4)	5.71 (2)
Dual user of cigarettes and e-cigarettes	8.28 (13)	6.56 (8)	14.29 (5)
*COVID-19 vaccine doses received*			
Not vaccinated^1^	22.3 (35)	-	100 (35)
At least one dose^2^	77.71 (122)	100 (122)	-
Fully Vaccinated^3^	97.54 (119)	97.54 (119)	-

*n = 156 for ‘Hispanic and Latino’ due to missing data

Vaccine classifications are as follows

^1^Not vaccinated (0 dose)

^2^Atleast one dose (1 dose of J&J or 1 dose of Pfizer-BioNTech/Moderna)

^3^Fully Vaccinated (1 dose of J&J or 2 doses of Pfizer-BioNTech/Moderna)

### Reasons for receiving the COVID-19 vaccination

**Fig 2:**
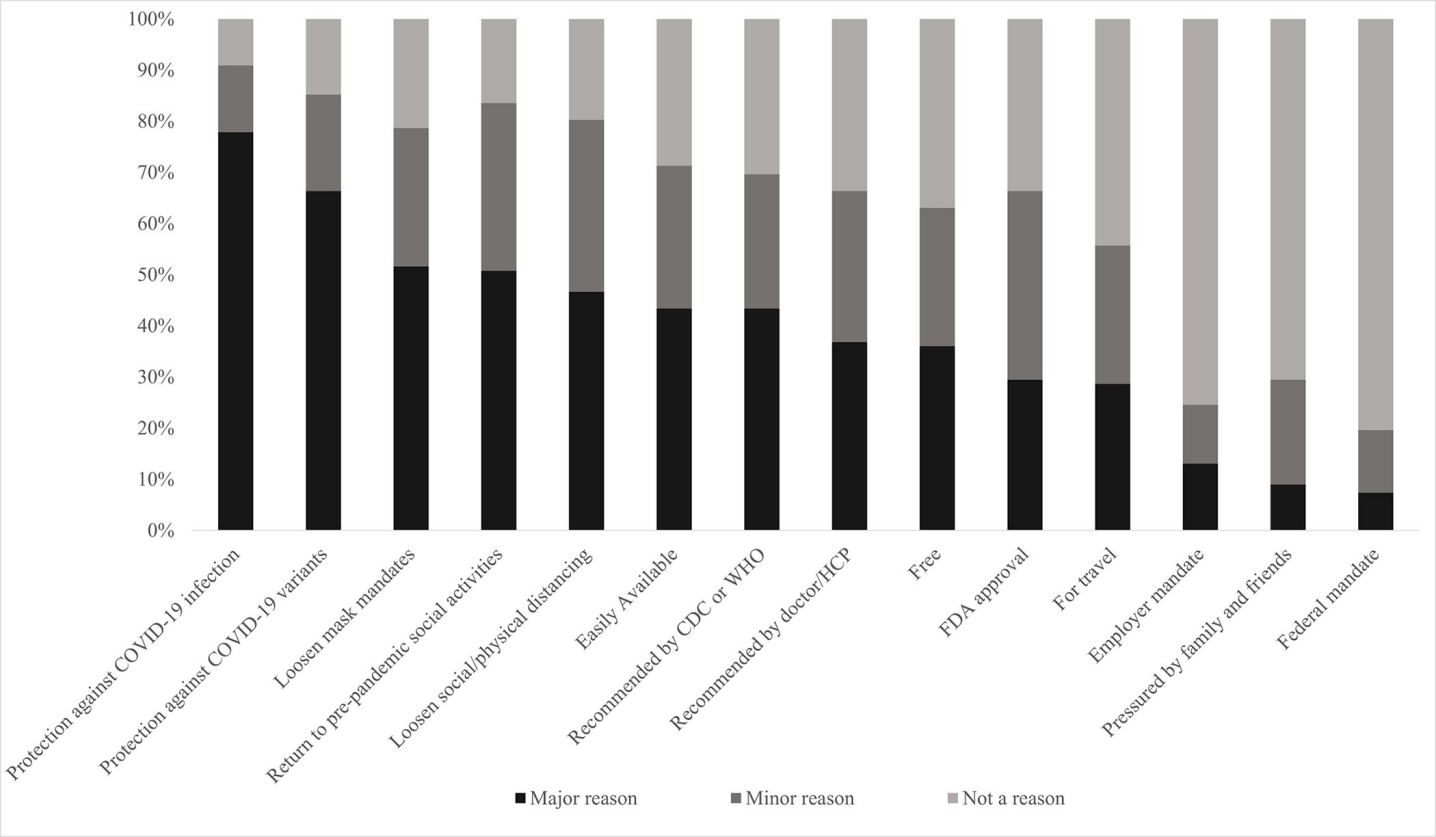
Reasons that prompted tobacco users to receive the COVID-19 vaccine (n = 122).

Of the participants (n = 122) that received at least one dose of the COVID-19 vaccine, the major reasons that prompted them to receive the COVID-19 vaccine were: to protect them against COVID-19 infection (77%), to protect them against COVID-19 variants (66%), and to loosen restrictions on mask mandates and social/physical distancing (51%) (Fig 2).

### Reasons for not receiving the COVID-19 vaccination

**Fig 3:**
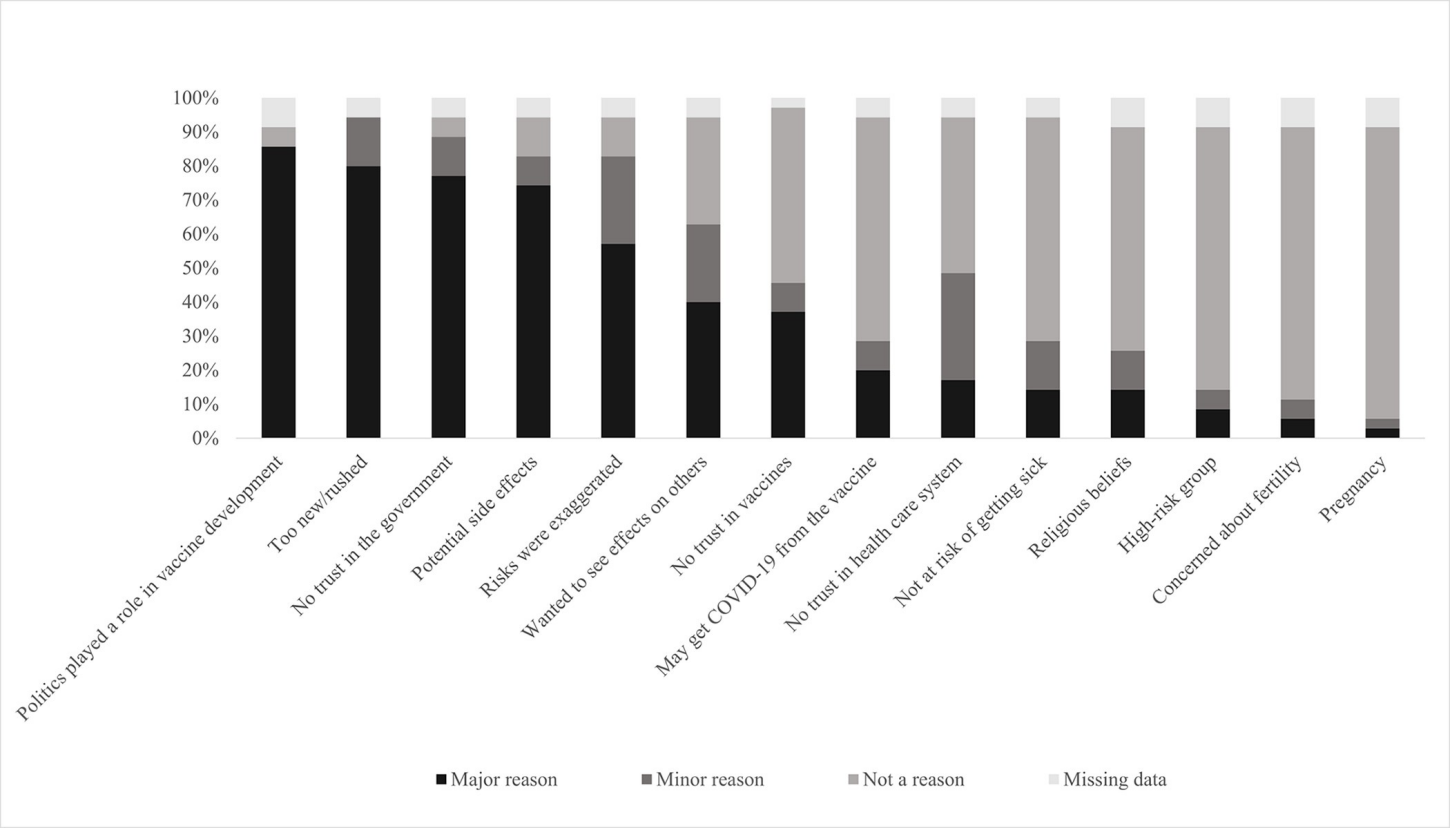
Reasons that prompted tobacco users to decline/choose not to receive the COVID-19 vaccine (n = 35).

From the 22% of participants who did not receive a COVID-19 vaccine, the major reasons they declined the COVID-19 vaccine were: they thought politics played too much of a role in the COVID-19 vaccine development process (94%), they felt the COVID-19 vaccine was too new/rushed (85%), and they did not trust the government to make sure the COVID-19 vaccine was safe and effective (82%) (Fig 3).

### Trust in individuals regarding the COVID-19 vaccine

**Fig 4:**
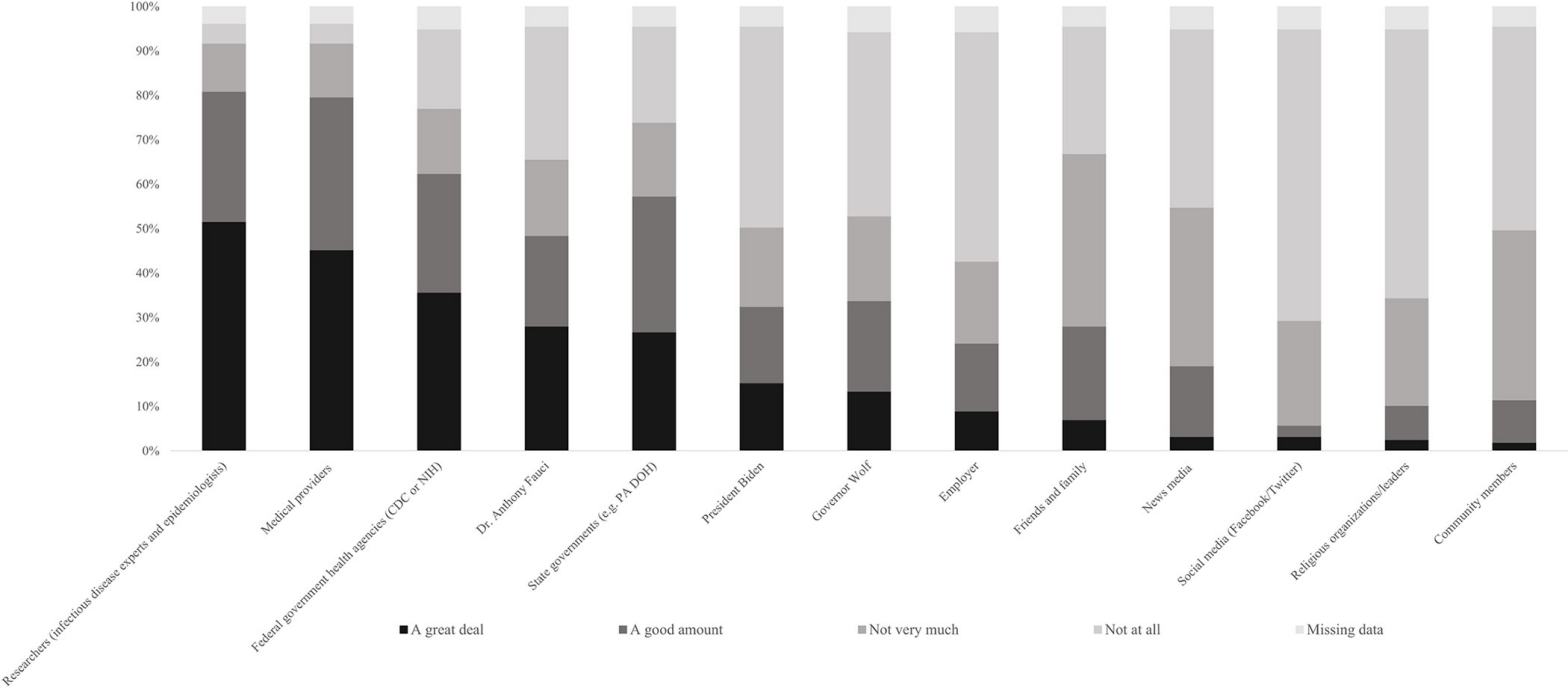
Tobacco users trust in the following people to advise them about the COVID-19 vaccine (n = 157).

People who tobacco users trusted a great deal to provide them with information regarding the COVID-19 vaccine were researchers who specialized in related topics (like epidemiologists) (53%), medical providers (such as doctors and nurses) (47%), and federal government health agencies (like CDC or NIH) (37%). Two-thirds of the participants mentioned not trusting social media such as Facebook or Twitter to advise them about the COVID-19 vaccine at all (Fig 4).

### Vaccine uptake and DOI theory

**Fig 5:**
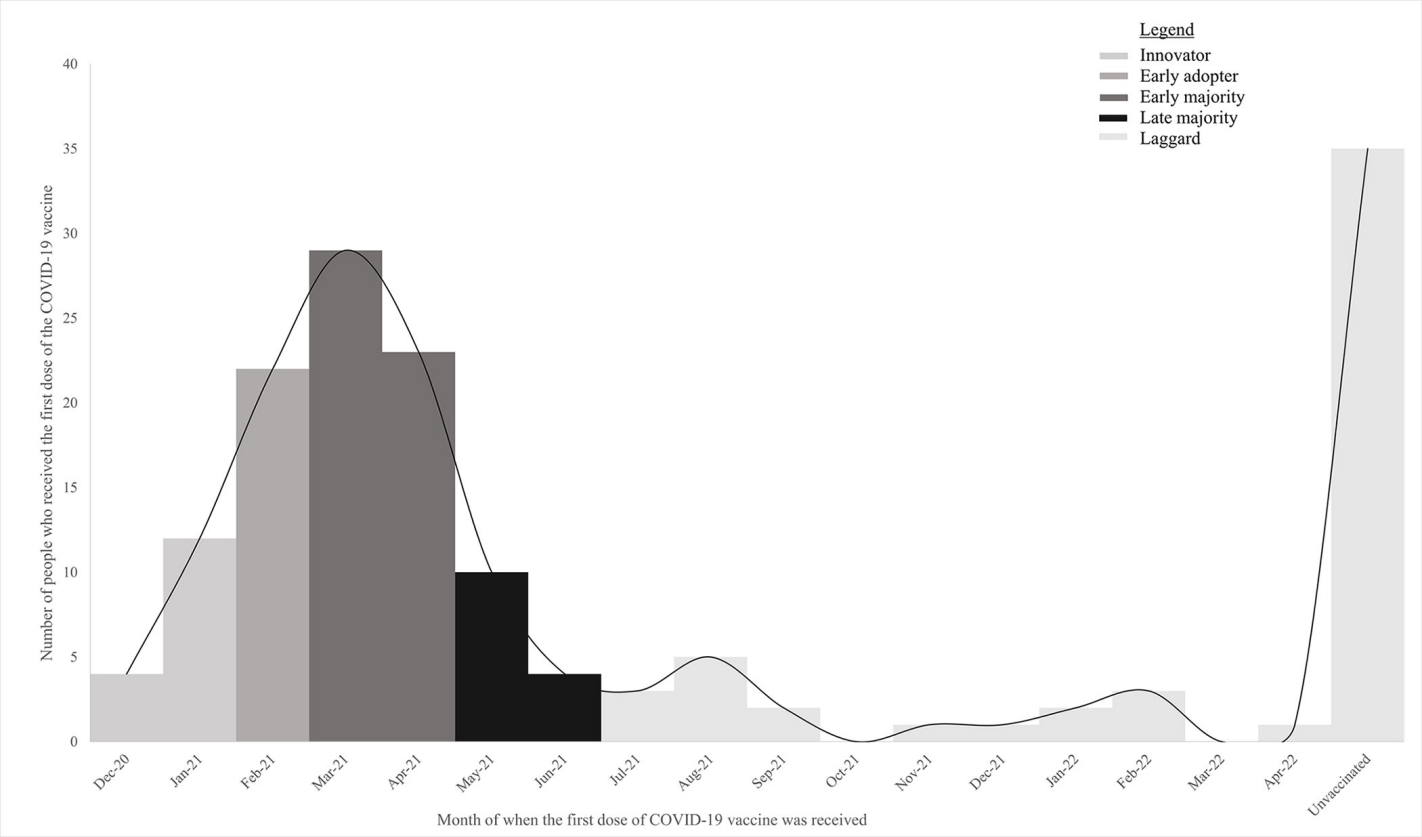
Applying the DOI theory to the month when people received the first dose of the COVID-19 vaccination (n = 157).

Nearly 78% of participants reported receiving at least one dose of a COVID-19 vaccine (Table 1). Among our study population, being male (*p* = 0.0213, OR = 5.608; 95% CI: 1.293–24.324), previously receiving an annual flu vaccine (*p* = 0.0136, OR = 4.530; 95% CI: 1.365–15.033) and trusting the federal government a great deal to advise tobacco users about the COVID-19 vaccine (*p* = < .0001, OR = 4.294; 95% CI: 2.396–7.698) were significant predictors of receiving at least one dose of the COVID-19 vaccine (Table 2). Using the DOI theory, vaccination uptake in our study population resulted in the five adopter categories as follows: 10% innovators, 14% early adopters, 33% early majority, 11% late majority, and 32% laggards (Fig 5). The only significant predictor of early vaccine uptake (innovator and early adopter) was loosening restrictions on mask mandates and social/physical distancing (*p* = 0.0180, OR = 2.237; 95% CI: 1.148–4.360) (Table 3).

**Table 2:** Logistic regression model with predictors of receiving at least one dose of the COVID-19 vaccine vs. not vaccinated.

Characteristic	Odds Ratio (OR)	95%Confidence Interval (CI)	P-value
Sex^a^	5.608	1.293–24.324	0.0213
Receive influenza vaccine each year^b^	4.530	1.365–15.033	0.0136
Trusts federal government^c^	4.294	2.396–7.698	< .0001

^a^Variable coded as 0 = female or other and 1 = male

^b^Variable coded as 0 = no and 1 = yes

^c^Variable caoded as 1 = Not at all, 2 = Not very much, 3 = A good amount, 4 = A great deal

**Table 3:** Logistic regression model with predictors of being an innovator or early adopter vs. other adopter categories.

Characteristic	Odds Ratio (OR)	95%Confidence Interval (CI)	P-value
Endorsed loosening restrictions on mask mandates and social/physical distancing^d^	2.237	1.148–4.360	0.0180

^d^Variable coded as 1 = not at all, 2 = minor reason, 3 = major reason

## Discussion

This study took an innovative approach at understanding COVID-19 vaccine uptake using the DOI theory. Application of the DOI theory has been seen in previous studies where the adoption of the influenza vaccine was mapped onto the DOI adopter categories to understand how pharmacist-delivered influenza vaccinations diffuse over time dependent on when they were allowed in various states [19]. Similar to the differences observed in other studies by adopter categorization, the DOI theory helped us reflect on the amount of time it took for adoption of a new idea, i.e., COVID-19 vaccine initiation in tobacco users, and how or why the idea gained acceptance over time.

Our study noted that during December 2020-January 2021, only 10% of tobacco users received the COVID-19 vaccine. Some may have received it due to employment as a HCW since they were able to receive the vaccine in December 2020 [12]. Other adults who use tobacco may have only been able to receive it after the addition of smokers to Phase 1a in January 2021 [8]. In comparison, the highest rates of COVID-19 vaccine uptake in our population were recorded in March 2021. This may be due to the availability of the COVID-19 vaccine to all aged sixteen and above by March 2021, and a time period of 3 months for tobacco users to observe vaccination effects in other people and gather information about the vaccine to make an informed decision [16, 20]. Additionally, March 8, 2021, was when the CDC announced that, “people who are fully vaccinated against COVID-19 can safely gather with other fully vaccinated people indoors without masks or social distancing” [21]. Finally, although a consistent decrease in COVID-19 vaccine uptake was noticed since March 2021, a small increase was observed in August 2021 that suggests tobacco users may have waited for the COVID-19 vaccine to be FDA-approved since full FDA approval (beyond emergency authorization) was acquired on August 23, 2021 [7]. Comparable to a new invention, since the COVID-19 was a new vaccine, the DOI theory also helped us identify characteristics of tobacco users who were more likely to be innovators or adopters and their motivation to receive the COVID-19 vaccine.

In our sample, tobacco users who were more likely to be innovators and early adopters were more likely to endorse loosening mask mandates as a major reason for receiving the COVID-19 vaccine. COVID-19 mask mandates began in PA on April 15^th^, 2020, and continued intermittently until June 28^th^, 2021 [12]. Having the mask mandate was important to reduce the spread of COVID-19 but some individuals were opposed to mask use because of physical discomfort, lack of perceived effectiveness and beliefs that masks were unnecessary in certain circumstances [22]. Specifically, tobacco users indicated being “less likely to smoke in public due to wearing a mask” [23]. Therefore, some tobacco users may have been motivated to receive the COVID-19 vaccine in the hopes of loosening mask mandates that restricted their ability to smoke in public.

Tobacco users who self-identified as male were also more likely to receive a dose of the COVID-19 vaccine than tobacco users who did not self-identify as male (OR = 6.576, 95% CI:1.524–28.381) [24, 25]. The reasoning behind gender differences regarding COVID-19 vaccine uptake is not clear, but some studies suggest that women of childbearing age and pregnant women may have concerns about side effects (such as infertility) and effects of the vaccine on their unborn child, although there is evidence that this does not occur [26–29]. A study assessing COVID-19 vaccine knowledge, attitude, acceptance, and hesitancy among pregnant and breastfeeding women found that fear of adverse events for the woman, child or both was the main reason for vaccine hesitancy [29]. Additionally, there was a concern among women regarding the COVID-19 vaccine potentially causing changes to their monthly cycle, but a recent study concluded that the COVID-19 vaccine is associated with a minor change in cycle length (which may be small and temporary), and no change in menses length [30]. In comparison, a study analyzing adherence to influenza vaccination during the 2018–2019 season concluded that females are more likely to receive the flu vaccination (62.8%) compared to their male counterparts (53.2%) [31].

Another predictor of COVID-19 vaccine uptake were tobacco users who have previously received an annual flu vaccine (OR = 4.4, 95% CI:1.33–14.6), which is consistent with other study findings. A study of over 1000 US adults that assessed intention to receive the COVID-19 vaccine found that those who received an influenza vaccine in the past year were associated with higher odds of intending to get the COVID-19 vaccine [32]. People who receive the flu shot may have increased trust and belief in perceived safety of vaccines, and may be more likely to get sick, thus being more likely to receive the COVID-19 vaccine as a preventative health measure vaccine [33, 34].

Finally, trusting the federal government a great deal to advise tobacco users about the COVID-19 vaccine (p = < .0001) was also a significant predictor of receiving at least one dose of the COVID-19 vaccine (Table 2). Previous research has also highlighted that trust in the government can affect public willingness to receive the COVID-19 vaccine [35]. A study of US adults from South Dakota concluded that trust in the government can increase COVID-19 vaccine uptake because if people trust the government, then they will likely accept government endorsement of information about the vaccine and eventually receive a vaccine [36].

Major reasons that led to people getting the vaccine were protection against COVID-19 infection, COVID-19 variants, and to loosen mask mandates. These reasons are consistent with previous public announcements made by the CDC and World Health Organization (WHO) whereby positively framing the benefits of getting the COVID-19 vaccine [37]. Advertisements that stated, “receiving the COVID-19 vaccine will protect you and your family from COVID-19”, was an effective communication strategy to promote COVID-19 vaccine uptake [38]. Additionally, due to research stating tobacco users were at a higher risk of suffering worse outcomes from COVID-19, tobacco users may have wanted to receive the vaccine to protect themselves from the virus [3, 4]. We recorded 75% of our study population (as of May 2022) fully vaccinated against COVID-19 which is close to the national average of 79% of the population ≥ 18 years of age (as of March 2023) who have completed a primary series of the COVID-19 vaccine [39]. Our study highlights an increase in uptake of COVID-19 vaccinations in tobacco users compared to data from a similar study of PA tobacco users in 2021 where 40% of tobacco users had not received their first dose of the COVID-19 vaccine despite being eligible for the vaccine for more than 3 months [10]. Nonetheless, our study data was recorded in 2022 when the COVID-19 vaccine had been available for more than a year but 22% were still not vaccinated for COVID-19. This finding highlights the prevalence of COVID-19 vaccine hesitancy in our population, with our unvaccinated rates similar to the national average of 21% [39].

Factors recorded as reasons for declining the COVID-19 vaccine by tobacco users in this study were: (a) politics play too much of a role in the COVID-19 vaccine development process, (b) COVID-19 vaccine was too new/rushed, and (c) they did not trust the government to make sure the COVID-19 vaccine was safe and effective. These outcomes relate to the current literature regarding politics during the COVID-19 era as studies have shown that people who identify with the Democratic party were more likely to receive the COVID-19 vaccine, as opposed to their Republican counterparts [40]. Previous studies have also highlighted that political parties mostly aligned with conservative voters (such as the Republican party in the US) lean towards opposing and generally undermining the legitimacy of what they perceive to be progressive authorities such as academics, scientists, and some governmental organizations such as the CDC or FDA [38, 40, 41]. These beliefs may have played a role in tobacco users’ beliefs regarding the COVID-19 vaccine and trust in the government regarding the safety of the vaccine. To improve tobacco user beliefs about the COVID-19 vaccine in the future, the government should aim to communicate the safety and efficacy of vaccines effectively, reduce profound political divisions, and restore trust in science and political parties [41]. In states where there are local government officials (e.g., State governors, or city mayors) who are from a different political party from the federal government in power, it may be particularly important for those local officials to publicly support vaccination. Similarly, it may be important for local health officials to prominently encourage vaccination, as they may have more impact on people who do not trust federal public health authorities.

Trust played a big role in tobacco users’ opinions about the COVID-19 vaccine. Researchers, medical providers and federal government health agencies were highlighted as people who tobacco users trusted a great deal to advise them about the COVID-19 vaccine. A study analyzing public trust in different agents related to the prevention of COVID-19 concluded that trust in the scientific community played the most fundamental role in receiving the COVID-19 vaccine [42]. Since COVID-19 was a new disease in 2020 and people were learning new information every day, it is likely that tobacco users may have felt that they trusted those who review empirical evidence, such as researchers, to provide them with the best and most up-to-date knowledge regarding the vaccine [42]. This finding differs from previous studies where U.S. residents tend to trust medical doctors more than researchers generally because doctors provide direct care and recommendations to them [43]. It is also important to note that this sample was recruited from a tobacco research registry and respondents may have had more positive views of research than the general public. Finally, as COVID-19 spread in 2020 and 2021, federal health government agencies such as the CDC and NIH were some of the most reliable sources that had access to new information about COVID-19. The CDC posted up-to-date information about COVID-19 cases, deaths, vaccinations, as well as information on precautions, symptoms, and treatment of COVID-19 [39]. Knowing that there is lack of tobacco users’ trust in the federal governments (37%), the government should focus on communicating the most up-to-date information to the public in the event of a future pandemic as communication can lead to increased trust in these organizations.

Limitations were present for this study. This study is non-representative of the entire US population and surveyed tobacco users only in one state (PA) and who were part of an existing research registry. Nonetheless, the survey captured information from participants after the COVID-19 vaccine had been available for more than a year so everyone had the opportunity to receive the vaccine. Our study had a small sample size, and was cross-sectional in nature, so causality cannot be inferred from our data. Some participants (such as healthcare workers) may have received a mandate from their employers to be vaccinated as a condition of their employment, and that may have led them to receiving the COVID-19 vaccine. Thus, we cannot infer that all participants who were categorized as innovators or early adopters was because they all wanted to receive the COVID-19 vaccine earlier than others. Finally, our study only collected quantitative data to gain preliminary insight into how tobacco users felt about the COVID-19 vaccine. Moving forward, open-ended qualitative questions using semi-structured interviews can be more helpful in understanding why tobacco users felt a certain way about the COVID-19 vaccine and how the vaccine has impacted their lives. Future studies can explore the major reasons in depth on why tobacco users received or did not receive the COVID-19 vaccine and the policy, community, organizational, interpersonal, and individual factors that influenced them to do so. A future qualitative study can provide rich data to support our findings from this paper and gain insight into participants’ experiences and interpretations. This study also had some strengths. First, this is one of the first studies that applies the DOI theory to COVID-19 vaccine uptake. Previous studies have highlighted the DOI theory and its factors as a means to explain the influenza vaccination or a new product/design, but this theory has not been used to describe COVID-19 vaccine uptake yet. Second, data from this study can be used as a basis for longitudinal data collection of COVID-19 vaccine hesitancy among tobacco users for future studies, especially to compare and contrast COVID-19 booster vaccine uptake. Qualitative research in this area can also support this data and explore more factors for why participants feel a certain way towards the COVID-19 vaccine. Finally, this study can help policymakers understand factors that contribute to COVID-19 vaccine hesitancy so we can create tailored interventions in the future to improve vaccine uptake among tobacco users, and eventually the general public.

## Conclusion

Other studies have suggested that there will be future outbreaks of infectious diseases, demanding rapid development of safe and effective vaccines, similar to the COVID-19 vaccine [44–46]. Using the DOI theory, researchers can apply this concept to promote vaccine acceptability and uptake, and understand the motivations and beliefs behind people receiving or declining vaccines. Public health professionals may use this information to develop more targeted interventions in the future to improve COVID-19 and other vaccine uptake among tobacco users. The results of this study emphasize the importance of understanding factors that drive vaccine uptake and trust on advice provided by researchers and government health officials. Thus, it is critical that these officials communicate clearly with the public regarding spread of a disease, mask mandates, and rationale for the rapid approval of a vaccine. This way, when evidence on efficacy of a vaccine is confirmed, government officials can communicate this information to the public with full transparency.

## Supporting information

S1 Appendix (PDF)
