# Supplementary material for: Using the diffusion of innovations theory to understand factors associated with COVID-19 vaccination among tobacco users
Source: PLoS One. 2024 Dec 12;19(12):e0309780. doi: 10.1371/journal.pone.0309780 (PMC11637259; doi:10.1371/journal.pone.0309780)
Supplement: S1 Appendix — (PDF) [file pone.0309780.s001.pdf]

| # | Variable / Field Name | Field Label<br>*Field Note* | Field Attributes (Field Type, Validation, Choices, Calculations, etc.) |
|---|---|---|---|
| 1 | [record_id] | Record ID | text |
| 2 | [lang_choice] | Thank you for your interest. Before we begin, this survey is available in English and Spanish. Please choose the language below that you are most comfortable with. | radio, Required<br>1 English<br>2 Spanish/Espanol |
| 3 | [english]<br>Show the eld ONLY if:<br>[lang_choice] = '1' | You selected English. Please continue with the rest of the survey. | descriptive |
| 4 | [spanish]<br>Show the eld ONLY if:<br>[lang_choice] = '2' | You selected Spanish. Please click on the link below, which will take you to the Spanish survey.https://redcap.link/ rstlinePA_spanish | descriptive |
| 5 | [hcworker_screen] | Section Header: *Thank you for being willing to participate in our project. First, we will ask a few questions to see if you are eligible to complete the survey.*<br>Do you work in the eld of healthcare? | yesno, Required<br>1 Yes<br>0 No |
| 6 | [pa_screen] | Do you work in Pennsylvania? | yesno, Required<br>1 Yes<br>0 No |
| 7 | [end_screen1]<br>Show the eld ONLY if:<br>[hcworker_screen] = '0' or [pa_screen] = '0' | We are sorry, but you do not meet the criteria to participate in this survey. Thank you for your time. | descriptive |
| 8 | [healthcare_worker_survey_screen_complete] | Section Header: *Form Status*<br>Complete? | dropdown<br>0 Incomplete<br>1 Unveri ed<br>2 Complete |

| | | | |
|---|---|---|---|
| 122 | [intro_2]<br><br>Show the eld ONLY if:<br>[part2] = '1' | Great! Let's get started on the Penn State survey! After you complete this survey, you will be directed to a separate form to register for 2 opportunities to win a $250 gift card.First, we'd<br>like to understand more about your experiences with and feelings about the COVID-19 vaccine. | descriptive |
| 123 | [vaxdose]<br><br>Show the eld ONLY if:<br>[part2] = '1' | Have you received at least one dose of a COVID-19 vaccine? | yesno<br>1 Yes<br>0 No |
| 124 | [vaxtype]<br><br>Show the eld ONLY if:<br>[part2] = '1' and [vaxd ose] = '1' | Did you receive a vaccine that required only one shot or two shots spread out over a few weeks? (This does NOT refer to booster shots) | radio<br>1 One dose (Johnson & Johnson)<br>2 Two doses (P zer or Moderna)<br>3 Don't know |
| 125 | [vaxdate]<br><br>Show the eld ONLY if:<br>[part2] = '1' and [vaxt ype] = '1' | When did you receive the COVID-19 vaccine? Please provide an estimated month and year (This does NOT refer to booster shots) | radio<br>1 December 2020<br>2 January 2021<br>3 February 2021<br>4 March 2021<br>5 April 2021<br>6 May 2021<br>7 June 2021<br>8 July 2021<br>9 August 2021<br>10 September 2021<br>11 October 2021<br>12 November 2021<br>13 December 2021<br>14 January 2022<br>15 February 2022 |

| | | | |
|---|---|---|---|
| | | | 16 March 2022<br>17 April 2022<br>18 May 2022<br>19 June 2022<br>20 July 2022<br>21 August 2022<br>22 September 2022<br>23 October 2022<br>24 November 2022<br>25 December 2022<br>26 January 2023<br>27 February 2023 |
| 126 | [twodose]<br>Show the eld ONLY if:<br>[part2] = '1' and [vaxt ype] = '2' | Have you already received both of your doses of the COVID-19 vaccine? | radio, Required<br>1 Yes, I have already received both doses<br>2 No, but I am scheduled to receive the second dose<br>3 No, but I plan to receive the second dose<br>4 No, I have not decided if I will receive a second dose<br>5 No, I do not plan to receive a second dose |
| 127 | [no_twodose]<br>Show the eld ONLY if:<br>[part2] = '1' and [two dose] = '4' and [twod ose] = '5' | If you have not decided to/do not plan to receive the second dose of the COVID-19 vaccine, can you explain why? | notes |

| | | | |
|---|---|---|---|
| 128 | [first_dose]<br><br>Show the eld ONLY if:<br>[part2] = '1' and [vaxt ype] = '2' | When did you receive the rst dose of the COVID-19 vaccine? Please provide an estimated month and year (This does NOT refer to booster shots) | radio<br>1 December 2020<br>2 January 2021<br>3 February 2021<br>4 March 2021<br>5 April 2021<br>6 May 2021<br>7 June 2021<br>8 July 2021<br>9 August 2021<br>10 September 2021<br>11 October 2021<br>12 November 2021<br>13 December 2021<br>14 January 2022<br>15 February 2022<br>16 March 2022<br>17 April 2022<br>18 May 2022<br>19 June 2022<br>20 July 2022<br>21 August 2022<br>22 September 2022<br>23 October 2022<br>24 November 2022<br>25 December 2022 |

| | | | |
|---|---|---|---|
| | | | 26 January 2023<br>27 February 2023 |
| 129 | [second_dose]<br><br>Show the eld ONLY if:<br>[part2] = '1' and [vaxt ype] = '2' and [twodos e] = '1' | When did you receive the second dose of the COVID-19 vaccine? Please provide an estimated month and year (This does NOT refer to booster shots) | radio<br>1 December 2020<br>2 January 2021<br>3 February 2021<br>4 March 2021<br>5 April 2021<br>6 May 2021<br>7 June 2021<br>8 July 2021<br>9 August 2021<br>10 September 2021<br>11 October 2021<br>12 November 2021<br>13 December 2021<br>14 January 2022 |
| | | | 15 February 2022<br>16 March 2022<br>17 April 2022<br>18 May 2022<br>19 June 2022<br>20 July 2022<br>21 August 2022<br>22 September 2022<br>23 October 2022 |

| | | | |
|---|---|---|---|
| | | | 24 November 2022<br>25 December 2022<br>26 January 2023<br>27 February 2023 |
| 130 | [supportmand]<br><br>Show the eld ONLY if:<br>[part2] = '1' and [vaxdose] = '1' or [vaxdose] = '0' | Do you support or oppose your employer requiring all employees who work with patients to become vaccinated? | radio<br>1 Strongly support<br>2 Somewhat support<br>3 Somewhat oppose<br>4 Strongly oppose |
| 131 | [covinf]<br><br>Show the eld ONLY if:<br>[part2] = '1' and [vaxdose] = '1' | Section Header: *What prompted you to receive a COVID19 vaccine?*<br><br>To protect me against COVID-19 infection | radio (Matrix)<br>1 Major reason<br>2 Minor reason<br>3 Not at all |
| 132 | [covvar]<br><br>Show the eld ONLY if:<br>[part2] = '1' and [vaxdose] = '1' | To protect me against COVID-19 variants | radio (Matrix)<br>1 Major reason<br>2 Minor reason<br>3 Not at all |
| 133 | [socact]<br><br>Show the eld ONLY if:<br>[part2] = '1' and [vaxdose] = '1' | To return to pre-pandemic social activities | radio (Matrix)<br>1 Major reason<br>2 Minor reason<br>3 Not at all |
| 134 | [maskrest]<br><br>Show the eld ONLY if:<br>[part2] = '1' and [vaxdose] = '1' | To loosen restrictions on mask mandates | radio (Matrix)<br>1 Major reason<br>2 Minor reason<br>3 Not at all |

| | | | |
|---|---|---|---|
| 135 | [socrest]<br><br>Show the eld ONLY if:<br>[part2] = '1' and [vaxdose] = '1' | To loosen restrictions on social/physical distancing | radio (Matrix)<br>1 Major reason<br>2 Minor reason<br>3 Not at all |
| 136 | [travel]<br><br>Show the eld ONLY if:<br>[part2] = '1' and [vaxdose] = '1' | For travel purposes | radio (Matrix)<br>1 Major reason<br>2 Minor reason<br>3 Not at all |
| 137 | [vaxfree]<br><br>Show the eld ONLY if:<br>[part2] = '1' and [vaxdose] = '1' | Vaccine is free | radio (Matrix)<br>1 Major reason<br>2 Minor reason<br>3 Not at all |
| 138 | [vaxavail]<br><br>Show the eld ONLY if:<br>[part2] = '1' and [vaxdose] = '1' | Vaccine is easily available | radio (Matrix)<br>1 Major reason<br>2 Minor reason<br>3 Not at all |
| 139 | [recdoc]<br><br>Show the eld ONLY if:<br>[part2] = '1' and [vaxdose] = '1' | Recommendation by a doctor/healthcare professional | radio (Matrix)<br>1 Major reason<br>2 Minor reason<br>3 Not at all |
| 140 | [recgov]<br><br>Show the eld ONLY if:<br>[part2] = '1' and [vaxdose] = '1' | Recommendation by CDC, WHO, or other government organization(s) | radio (Matrix)<br>1 Major reason<br>2 Minor reason<br>3 Not at all |
| 141 | [pressure]<br><br>Show the eld ONLY if:<br>[part2] = '1' and [vaxdose] = '1' | Pressured by family and friends to get the vaccine | radio (Matrix)<br>1 Major reason<br>2 Minor reason<br>3 Not at all |

| | | | |
|---|---|---|---|
| 142 | [fedmand]<br><br>Show the eld ONLY if:<br>[part2] = '1' and [vaxdose] = '1' | Federal mandate | radio (Matrix)<br>1 Major reason<br>2 Minor reason<br>3 Not at all |
| 143 | [empmand]<br><br>Show the eld ONLY if:<br>[part2] = '1' and [vaxdose] = '1' | Employer mandate | radio (Matrix)<br>1 Major reason<br>2 Minor reason<br>3 Not at all |
| 144 | [approval] | FDA approval | radio (Matrix) |

| | | | |
|---|---|---|---|
| | Show the field ONLY if:<br>[part2] = '1' and [vaxdose] = '1' | | 1 Major reason<br>2 Minor reason<br>3 Not at all |
| 145 | [othervax]<br>Show the field ONLY if:<br>[part2] = '1' and [vaxdose] = '1' | Other reasons | radio (Matrix)<br>1 Major reason<br>2 Minor reason<br>3 Not at all |
| 146 | [other_vax]<br>Show the field ONLY if:<br>[part2] = '1' and [othervax] = '1' or [othervax] = '2' | If other, please explain: | notes |
| 147 | [unvaxpeople]<br>Show the field ONLY if:<br>[part2] = '1' | What are your views on unvaccinated people? (Check all that apply) | checkbox<br>10 unvaxpeople___10 Everyone has a right to their personal decisions, so I respect/support them<br>12 unvaxpeople___12 Since I am vaccinated, other people's vaccine choice doesn't affect me<br>14 unvaxpeople___14 I am okay with other people's vaccine choice, as long as they wear a mask and maintain social distance<br>16 unvaxpeople___16 They are putting people like me at risk<br>17 unvaxpeople___17 They are selfish and ignorant<br>18 unvaxpeople___18 They should not be allowed to work<br>19 unvaxpeople___19 I feel they are misled by misinformation |

| | | | |
|---|---|---|---|
| | | | 20 \| unvaxpeople___20 \| It makes me upset or angry<br>21 \| unvaxpeople___21 \| Other |
| 148 | [unvaxother]<br><br>Show the field ONLY if:<br>[part2] = '1' and [unvaxpeople(21)] = '1' | If other, please explain: | notes |
| 149 | [sideeffect]<br><br>Show the field ONLY if:<br>[part2] = '1' and [vaxdose] = '0' | Section Header: *Below are reasons why someone may not receive the COVID-19 vaccine. For the reasons listed below, please indicate whether you feel each is a major reason, minor reason, or not a reason for why you may decline/choose not to receive the COVID-19 vaccine.*<br><br>I'm worried about possible side effects | radio (Matrix)<br>1 \| Major reason<br>2 \| Minor reason<br>3 \| Not a reason |
| 150 | [trustgov]<br><br>Show the field ONLY if:<br>[part2] = '1' and [vaxdose] = '0' | I don't trust the government to make sure the COVID-19 vaccine is safe and effective | radio (Matrix)<br>1 \| Major reason<br>2 \| Minor reason<br>3 \| Not a reason |
| 151 | [toonew]<br><br>Show the field ONLY if:<br>[part2] = '1' and [vaxdose] = '0' | I feel that the COVID-19 vaccine is too new/rushed | radio (Matrix)<br>1 \| Major reason<br>2 \| Minor reason<br>3 \| Not a reason |
| 152 | [workonother]<br><br>Show the field ONLY if:<br>[part2] = '1' and [vaxdose] = '0' | I want to wait and see how it works for other people | radio (Matrix)<br>1 \| Major reason<br>2 \| Minor reason<br>3 \| Not a reason |
| 153 | [politics]<br><br>Show the field ONLY if:<br>[part2] = '1' and [vaxdose] = '0' | I think politics have played too much of a role in the COVID-19 vaccine development process | radio (Matrix)<br>1 \| Major reason<br>2 \| Minor reason<br>3 \| Not a reason |
| 154 | [covrisk]<br><br>Show the field ONLY if:<br>[part2] = '1' and [vaxdose] = '0' | I think the risks of COVID-19 are being exaggerated | radio (Matrix)<br>1 \| Major reason<br>2 \| Minor reason<br>3 \| Not a reason |
| 155 | [vaxtrust]<br><br>Show the field ONLY if:<br>[part2] = '1' and [vaxdose] = '0' | I don't trust vaccines in general | radio (Matrix)<br>1 \| Major reason<br>2 \| Minor reason<br>3 \| Not a reason |
| 156 | [healthtrust] | I don't trust the health care system | radio (Matrix) |

| | | | |
|---|---|---|---|
| | Show the eld ONLY if:<br>[part2] = '1' and [vaxd ose] = '0' | | 1 Major reason<br>2 Minor reason<br>3 Not a reason |
| 157 | [vaxcov]<br><br>Show the eld ONLY if:<br>[part2] = '1' and [vaxd ose] = '0' | I'm worried that I might get COVID-19 from the COVID-19 vaccine | radio (Matrix)<br>1 Major reason<br>2 Minor reason<br>3 Not a reason |
| 158 | [sickcov]<br><br>Show the eld ONLY if:<br>[part2] = '1' and [vaxd ose] = '0' | I don't think I'm at risk of getting sick with COVID-19 | radio (Matrix)<br>1 Major reason<br>2 Minor reason<br>3 Not a reason |
| 159 | [highrisk]<br><br>Show the eld ONLY if:<br>[part2] = '1' and [vaxd ose] = '0' | I am considered a high-risk group for getting the COVID-19 vaccine (e.g., I have an allergy to vaccine ingredients, immunocompromised, etc.) | radio (Matrix)<br>1 Major reason<br>2 Minor reason<br>3 Not a reason |
| 160 | [pregnant]<br><br>Show the eld ONLY if:<br>[part2] = '1' and [vaxd ose] = '0' | I am pregnant, trying to get pregnant, or breastfeeding | radio (Matrix)<br>1 Major reason<br>2 Minor reason<br>3 Not a reason |
| 161 | [fertility]<br><br>Show the eld ONLY if:<br>[part2] = '1' and [vaxd ose] = '0' | I am concerned about miscarriage or fertility issues in the future | radio (Matrix)<br>1 Major reason<br>2 Minor reason<br>3 Not a reason |
| 162 | [religious]<br><br>Show the eld ONLY if:<br>[part2] = '1' and [vaxd ose] = '0' | I cannot get the COVID-19 vaccine due to religious beliefs | radio (Matrix)<br>1 Major reason<br>2 Minor reason<br>3 Not a reason |
| 163 | [other]<br><br>Show the eld ONLY if: | Other reasons | radio (Matrix)<br>1 Major reason |

| | | | |
|---|---|---|---|
| | [part2] = '1' and [vaxd ose] = '0' | | 2 Minor reason<br>3 Not a reason |
| 164 | [other_novax]<br><br>Show the eld ONLY if:<br>[part2] = '1' and [othe r] = '1' or [other] = '2' | If other, please explain: | notes |

| | | | |
|---|---|---|---|
| 165 | [boostershot]<br><br>Show the eld ONLY if:<br>[part2] = '1' and [vaxd ose] = '1' | Section Header: *COVID-19 Booster shots The next few questions inquire about the COVID-19 booster shots.*<br><br>The FDA has authorized COVID-19 vaccine booster shots and recommends a booster dose to individuals ≥ 5 years of age. Have you personally received the COVID-19 booster shot? | radio<br>1 Yes, I have received the COVID-19 booster shot<br>2 No, I have scheduled my COVID-19 booster shot<br>3 No, I plan to schedule my COVID-19 booster shot in the near future<br>4 No, it has not been long enough since my primary COVID-19 vaccination<br>5 No, I do not plan on receiving to receive the COVID-19 booster shot |
| 166 | [boosterdate]<br><br>Show the eld ONLY if:<br>[part2] = '1' and [boos tershot] = '1' and [vax dose] = '1' | When did you receive the COVID-19 booster shot? Please provide an estimated month and year | radio<br>1 August 2021<br>2 September 2021<br>3 October 2021<br>4 November 2021<br>5 December 2021<br>6 January 2022<br>7 February 2022<br>8 March 2022<br>9 April 2022<br>10 May 2022<br>11 June 2022 |

| | | | |
|---|---|---|---|
| | | | 12 July 2022<br>13 August 2022<br>14 September 2022<br>15 October 2022<br>16 November 2022<br>17 December 2022<br>18 January 2023<br>19 February 2023 |
| 167 | [bsick]<br><br>Show the eld ONLY if:<br>[part2] = '1' and [boostershot] = '5' | Section Header: *Below are reasons why someone might not want to get the booster shot. Please indicate whether each is a major reason, minor reason, or not a reason why you might not get the booster shot vaccine.*<br><br>I experienced e ects after receiving the COVID-19 vaccine (i.e., fever, chills, aches) | radio (Matrix)<br>1 Major reason<br>2 Minor reason<br>3 Not a reason |
| 168 | [btoonew]<br><br>Show the eld ONLY if:<br>[part2] = '1' and [boostershot] = '5' | I feel that the booster shot is too new | radio (Matrix)<br>1 Major reason<br>2 Minor reason<br>3 Not a reason |
| 169 | [bwait]<br><br>Show the eld ONLY if:<br>[part2] = '1' and [boostershot] = '5' | I want to wait and see how the booster shot works for other people | radio (Matrix)<br>1 Major reason<br>2 Minor reason<br>3 Not a reason |
| 170 | [bexaggerate]<br><br>Show the eld ONLY if:<br>[part2] = '1' and [boostershot] = '5' | I think the risks of COVID-19 are being exaggerated | radio (Matrix)<br>1 Major reason<br>2 Minor reason<br>3 Not a reason |
| 171 | [bgetcovid]<br><br>Show the eld ONLY if: | I'm worried that I might get COVID-19 from the booster shot | radio (Matrix)<br>1 Major reason |

| | | | |
|---|---|---|---|
| | [part2] = '1' and [boostershot] = '5' | | 2 Minor reason<br>3 Not a reason |
| 172 | [bnotsick]<br><br>Show the eld ONLY if:<br>[part2] = '1' and [boostershot] = '5' | I don't think I'm at risk of getting sick from COVID-19 | radio (Matrix)<br>1 Major reason<br>2 Minor reason<br>3 Not a reason |
| 173 | [bhighrisk]<br><br>Show the eld ONLY if:<br>[part2] = '1' and [boostershot] = '5' | I am not at a high-risk for getting COVID-19 | radio (Matrix)<br>1 Major reason<br>2 Minor reason<br>3 Not a reason |
| 174 | [ballergy]<br><br>Show the eld ONLY if:<br>[part2] = '1' and [boostershot] = '5' | I have an allergy to vaccine ingredients, immunocompromised, etc. | radio (Matrix)<br>1 Major reason<br>2 Minor reason<br>3 Not a reason |
| 175 | [bcovvar]<br><br>Show the eld ONLY if:<br>[part2] = '1' and [boostershot] = '5' | I would like to wait for new vaccine booster shots that better cover new variants | radio (Matrix)<br>1 Major reason<br>2 Minor reason<br>3 Not a reason |
| 176 | [bpregnant]<br><br>Show the eld ONLY if:<br>[part2] = '1' and [boostershot] = '5' | I am pregnant, trying to get pregnant, or breastfeeding | radio (Matrix)<br>1 Major reason<br>2 Minor reason<br>3 Not a reason |

| | | | |
|---|---|---|---|
| 177 | [bfertility]<br><br>Show the eld ONLY if:<br>[part2] = '1' and [boostershot] = '5' | I am concerned about miscarriage or fertility issues in the future | radio (Matrix)<br>1 Major reason<br>2 Minor reason<br>3 Not a reason |
| 178 | [breligion]<br><br>Show the eld ONLY if: | I cannot get the booster shot due to religious beliefs | radio (Matrix)<br>1 Major reason |

| | | | |
|---|---|---|---|
| | [part2] = '1' and [boostershot] = '5' | | 2 Minor reason<br>3 Not a reason |
| 179 | [otherbooster]<br><br>Show the eld ONLY if:<br>[part2] = '1' and [boostershot] = '5' | Other reasons | radio (Matrix)<br>1 Major reason<br>2 Minor reason<br>3 Not a reason |
| 180 | [other_nobooster]<br><br>Show the eld ONLY if:<br>[part2] = '1' and [otherbooster] = '2' or [otherbooster] = '1' | If other, please explain: | notes |
| 181 | [secondbooster]<br><br>Show the eld ONLY if:<br>[part2] = '1' and [boostershot] = '1' | Section Header: *Second COVID-19 Booster shots*<br><br>The FDA has authorized second COVID-19 vaccine booster shots and the CDC recommends a second booster dose to individuals 65 and older and those 50 and older with underlying medical conditions that increase their risk for severe disease from COVID-19. Have you personally received the second COVID-19 booster shot? | radio<br>1 Yes, I have received the second COVID-19 booster shot<br>2 No, I have not received the second COVID-19 booster shot but I have scheduled my second COVID-19 booster shot<br>3 No, I plan to schedule my second COVID-19 booster shot in the near future<br>4 No, it has not been long enough since my primary COVID-19 booster<br>5 No, I don't plan to receive the second booster shot at this time |
| 182 | [secboosterdate]<br><br>Show the eld ONLY if:<br>[part2] = '1' and [boostershot] = '1' and [vaxdose] = '1' | When did you receive the second COVID-19 booster shot? Please provide an estimated month and year | radio<br>1 March 2022<br>2 April 2022<br>3 May 2022<br>4 June 2022<br>5 July 2022 |

| | | | |
|---|---|---|---|
| | | | 6 August 2022 |
| | | | 7 September 2022<br>8 October 2022<br>9 November 2022<br>10 December 2022<br>11 January 2023<br>12 February 2023 |
| 183 | [noboosterreason]<br><br>Show the eld ONLY if:<br>[secondbooster] = '5' | If you do not plan to receive the second booster dose, can you explain why? | notes |
| 184 | [children] | Section Header: *COVID-19 vaccinations for children*<br><br>Do you have any children under the age of 18? (Select all that apply) | checkbox<br>1 children___1 No<br>2 children___2 Yes- aged 12-18<br>3 children___3 Yes- aged 5-11<br>4 children___4 Yes- less than 5 |
| 185 | [childvax]<br><br>Show the eld ONLY if:<br>[children(2)] = '1' | The P zer vaccine has been approved for children 12 years old+ since May 2021. Have your children received the COVID-19 vaccine? | radio<br>1 Yes, they have already completed the series<br>2 Yes, but they have only received the rst dose, and have scheduled their second dose |

| | | | |
|---|---|---|---|
| | | | 3 Yes, but they have only received the rst dose, and will not be receiving the second dose<br>4 No, but I scheduled their rst dose<br>5 No, I have not decided if my children will be getting the COVID19 vaccine<br>6 No, I do not plan to vaccinate my children |
| 186 | [youngchildvax]<br><br>Show the eld ONLY if:<br>[children(3)] = '1' | The P zer vaccine has been approved in children 5-11 years old since November 2021. Have your children received the COVID-19 vaccine? | radio<br>1 Yes, they have already completed the series<br>2 Yes, but they have only received the rst dose<br>3 No, but I scheduled their rst dose<br>4 No, I have not decided if my children will be getting the COVID19 vaccine<br>5 No, I do not plan to vaccinate my children |

| | | | |
|---|---|---|---|
| 187 | [recchildvax]<br><br>Show the eld ONLY if:<br>[children(4)] = '1' | The P zer vaccine has been approved in children under 5 years old since May 2022. Have your children received the COVID-19 vaccine? | radio<br>1 Yes, they have already completed the series<br>2 Yes, but they have only received the rst dose<br>3 No, but I scheduled their rst dose<br>4 No, I have not decided if my children will be getting the COVID19 vaccine<br>5 No, I do not plan to vaccinate my children |
| 188 | | | radio |

| | | | |
|---|---|---|---|
| | [recvax]<br><br>Show the eld ONLY if:<br>[children(1)] = '1' | COVID-19 vaccines have been approved for children 0-18 years of age. Would you recommend the COVID-19 vaccine to parents with children in this age range? | 1 Yes, I would recommend the COVID-19 vaccine to children ages 0-18<br>2 Yes, I would recommend the COVID-19 vaccine to ONLY children ages 5-18<br>3 No, I would not recommend the COVID-19 vaccine to children ages 0-18<br>4 I don't know |
| 189 | [childsideff]<br><br>Show the eld ONLY if:<br>[childvax] = '5' or [childvax] = '6' or [youngchildvax] = '4' or [youngchildvax] = '5' or [recchildvax] = '2' or [recchildvax] = '3' | Section Header: *Below are reasons why someone might not want to vaccinate their child against COVID-19. For the reasons listed below, please indicate whether you feel each is a major reason, minor reason, or not a reason for why you may decline/not recommend the COVID-19 vaccine/choose not to vaccinate your child against COVID19.*<br><br>I'm worried about possible side e ects in the future for children | radio (Matrix)<br>1 Major reason<br>2 Minor reason<br>3 Not a reason |
| 190 | [childrxn]<br><br>Show the eld ONLY if:<br>[childvax] = '5' or [childvax] = '6' or [youngchildvax] = '4' or [youngchildvax] = '5' or [recchildvax] = '2' or [recchildvax] = '3' | I (or a friend/family member) had a severe reaction to the COVID-19 vaccine | radio (Matrix)<br>1 Major reason<br>2 Minor reason<br>3 Not a reason |
| 191 | [childtrustgov]<br><br>Show the eld ONLY if:<br>[childvax] = '5' or [childvax] = '6' or [youngchildvax] = '4' or [youngchildvax] = '5' or [recchildvax] = '2' or [recchildvax] = '3' | I don't trust the government to make sure the COVID-19 vaccine is safe and e ective for children | radio (Matrix)<br>1 Major reason<br>2 Minor reason<br>3 Not a reason |

| | | | |
|---|---|---|---|
| 192 | [childtoonew]<br><br>Show the eld ONLY if:<br>[childvax] = '5' or [chil<br>dvax] = '6' or [youngc<br>hildvax] = '4' or [youn<br>gchildvax] = '5' or [rec<br>childvax] = '2' or [recc<br>hildvax] = '3' | I think the COVID-19 vaccine is too new | radio (Matrix)<br>1 Major reason<br>2 Minor reason<br>3 Not a reason |
| 193 | [otherchild]<br><br>Show the eld ONLY if:<br>[childvax] = '5' or [chil<br>dvax] = '6' or [youngc<br>hildvax] = '4' or [youn<br>gchildvax] = '5' or [rec<br>childvax] = '2' or [recc<br>hildvax] = '3' | I want to wait and see how it works on other children | radio (Matrix)<br>1 Major reason<br>2 Minor reason<br>3 Not a reason |
| 194 | [childpol]<br><br>Show the eld ONLY if:<br>[childvax] = '5' or [chil<br>dvax] = '6' or [youngc<br>hildvax] = '4' or [youn<br>gchildvax] = '5' or [rec<br>childvax] = '2' or [recc<br>hildvax] = '3' | I think politics has played too much of a role in the COVID-19 vaccine development process | radio (Matrix)<br>1 Major reason<br>2 Minor reason<br>3 Not a reason |
| 195 | [childrisk]<br><br>Show the eld ONLY if:<br>[childvax] = '5' or [chil<br>dvax] = '6' or [youngc<br>hildvax] = '4' or [youn<br>gchildvax] = '5' or [rec<br>childvax] = '2' or [recc<br>hildvax] = '3' | I think the risks of COVID-19 are being exaggerated | radio (Matrix)<br>1 Major reason<br>2 Minor reason<br>3 Not a reason |
| 196 | [childtrustvax]<br><br>Show the eld ONLY if:<br>[childvax] = '5' or [chil<br>dvax] = '6' or [youngc<br>hildvax] = '4' or [youn<br>gchildvax] = '5' or [rec<br>childvax] = '2' or [recc<br>hildvax] = '3' | I don't trust vaccines in general | radio (Matrix)<br>1 Major reason<br>2 Minor reason<br>3 Not a reason |

| | | | |
|---|---|---|---|
| 197 | [healthsystem]<br><br>Show the eld ONLY if:<br>[childvax] = '5' or [chil dvax] = '6' or [youngc hildvax] = '4' or [youn gchildvax] = '5' or [rec childvax] = '2' or [recc hildvax] = '3' | I don't trust the health care system | radio (Matrix)<br>1 Major reason<br>2 Minor reason<br>3 Not a reason |
| 198 | [getcov]<br><br>Show the eld ONLY if:<br>[childvax] = '5' or [chil dvax] = '6' or [youngc hildvax] = '4' or [youn gchildvax] = '5' or [rec childvax] = '2' or [recc hildvax] = '3' | I'm worried children may get COVID-19 from the vaccine | radio (Matrix)<br>1 Major reason<br>2 Minor reason<br>3 Not a reason |
| 199 | [notsick]<br><br>Show the eld ONLY if:<br>[childvax] = '5' or [chil dvax] = '6' or [youngc hildvax] = '4' or [youn gchildvax] = '5' or [rec childvax] = '2' or [recc hildvax] = '3' | I don't think children are at risk of getting sick from COVID-19 | radio (Matrix)<br>1 Major reason<br>2 Minor reason<br>3 Not a reason |
| 200 | [childhealthy]<br><br>Show the eld ONLY if:<br>[childvax] = '5' or [chil dvax] = '6' or [youngc hildvax] = '4' or [youn gchildvax] = '5' or [rec childvax] = '2' or [recc hildvax] = '3' | I think children are generally healthier and do not need the COVID-19 vaccine | radio (Matrix)<br>1 Major reason<br>2 Minor reason<br>3 Not a reason |
| 201 | [childreligious]<br><br>Show the eld ONLY if:<br>[childvax] = '5' or [chil dvax] = '6' or [youngc hildvax] = '4' or [youn gchildvax] = '5' or [rec childvax] = '2' or [recc hildvax] = '3' | Religious reasons | radio (Matrix)<br>1 Major reason<br>2 Minor reason<br>3 Not a reason |

| | | | |
|---|---|---|---|
| 202 | [childother]<br><br>Show the eld ONLY if:<br>[childvax] = '5' or [chil dvax] = '6' or [youngc hildvax] = '4' or [youn gchildvax] = '5' or [rec childvax] = '2' or [recc hildvax] = '3' | Other reasons | radio (Matrix)<br>1 Major reason<br>2 Minor reason<br>3 Not a reason |
| 203 | [other_nochildvax]<br><br>Show the eld ONLY if:<br>[childother] = '1' or [c hildother] = '2' | If other, please explain: | notes |
| 204 | [president]<br><br>Show the eld ONLY if:<br>[part2] = '1' and [vaxd ose] = '1' or [vaxdose] = '0' | Section Header: *How much do you trust each of the following to advise you about the COVID-19 vaccine?*<br><br>President Biden | radio (Matrix)<br>1 A great deal<br>2 A good amount<br>3 Not very much<br>4 Not at all |
| 205 | [drfauci]<br><br>Show the eld ONLY if:<br>[part2] = '1' and [vaxd ose] = '1' or [vaxdose] = '0' | Dr. Anthony Fauci | radio (Matrix)<br>1 A great deal<br>2 A good amount<br>3 Not very much<br>4 Not at all |
| 206 | [govwolf]<br><br>Show the eld ONLY if:<br>[part2] = '1' and [vaxd ose] = '1' or [vaxdose] = '0' | Governor Wolf | radio (Matrix)<br>1 A great deal<br>2 A good amount<br>3 Not very much<br>4 Not at all |
| 207 | [fedgovt]<br><br>Show the eld ONLY if:<br>[part2] = '1' and [vaxd ose] = '1' or [vaxdose] = '0' | Federal government health agencies, such as the Centers for Disease Control (CDC) or the National Institutes for Health (NIH) | radio (Matrix)<br>1 A great deal<br>2 A good amount<br>3 Not very much |

| | | | |
|---|---|---|---|
| | | | 4 Not at all |
| 208 | [stategovt] Show the eld ONLY if: [part2] = '1' and [vaxdose] = '1' or [vaxdose] = '0' | State government health agencies, such as the PA Department of Health | radio (Matrix)<br>1 A great deal<br>2 A good amount<br>3 Not very much<br>4 Not at all |
| 209 | [medprov] Show the eld ONLY if: [part2] = '1' and [vaxdose] = '1' or [vaxdose] = '0' | Medical providers, such as doctors and nurses | radio (Matrix)<br>1 A great deal<br>2 A good amount<br>3 Not very much<br>4 Not at all |
| 210 | [researchers] Show the eld ONLY if: [part2] = '1' and [vaxdose] = '1' or [vaxdose] = '0' | Researchers who specialize in related topics, such as infectious disease experts and epidemiologists | radio (Matrix)<br>1 A great deal<br>2 A good amount<br>3 Not very much<br>4 Not at all |
| 211 | [newsmedia] Show the eld ONLY if: [part2] = '1' and [vaxdose] = '1' or [vaxdose] = '0' | The news media such as national TV news, local TV news, newspapers | radio (Matrix)<br>1 A great deal<br>2 A good amount<br>3 Not very much<br>4 Not at all |
| 212 | [friends] Show the eld ONLY if: [part2] = '1' and [vaxdose] = '1' or [vaxdose] = '0' | Friends and family | radio (Matrix)<br>1 A great deal<br>2 A good amount<br>3 Not very much<br>4 Not at all |
| 213 | [socmedia] Show the eld ONLY if: [part2] = '1' and [vaxdose] = '1' or [vaxdose] | Social media such as Facebook or Twitter | radio (Matrix)<br>1 A great deal<br>2 A good amount |

| | | | |
|---|---|---|---|
| | = '0' | | 3 Not very much<br>4 Not at all |
| 214 | [community]<br><br>Show the eld ONLY if: [part2] = '1' and [vaxd ose] = '1' or [vaxdose] = '0' | Community members | radio (Matrix)<br>1 A great deal<br>2 A good amount<br>3 Not very much<br>4 Not at all |
| 215 | [religiousorgs]<br><br>Show the eld ONLY if: [part2] = '1' and [vaxd ose] = '1' or [vaxdose] = '0' | Religious organizations or their leaders | radio (Matrix)<br>1 A great deal<br>2 A good amount<br>3 Not very much<br>4 Not at all |
| 216 | [employer]<br><br>Show the eld ONLY if: [part2] = '1' and [vaxd ose] = '1' or [vaxdose] = '0' | My employer | radio (Matrix)<br>1 A great deal<br>2 A good amount<br>3 Not very much<br>4 Not at all |
| 217 | [cdc1] | Section Header: *CDC (Centers for Disease Control and Prevention) is the nation's public health agency that protects people by preventing and controlling disease, injury, disability, and responds when these health threats arise.*<br><br>How has your view of the CDC changed based on the agency's management of the COVID-19 pandemic?<br><br>My view is... | radio<br>1 More positive<br>2 Positive<br>3 Has not changed<br>4 Negative<br>5 More negative |
| 218 | [cdc2]<br><br>Show the eld ONLY if: [cdc1] = '1' or [cdc1] = '2' or [cdc1] = '4' or [c dc1] = '5' | In one or two sentences, can you tell us why your view of the CDC changed based on the agency's management of the COVID-19 pandemic? | text |
| 219 | [cdc3] | | radio |

| | | | |
|---|---|---|---|
| | | How has your view of the CDC changed based on the agency's COVID-19 vaccine recommendations?<br><br>My view is... | 1 More positive<br>2 Positive<br>3 Has not changed<br>4 Negative<br>5 More negative |
| 220 | [cdc4]<br><br>Show the eld ONLY if:<br>[cdc3] = '1' or [cdc3] = '2' or [cdc3] = '4' or [c dc3] = '5' | In one or two sentences, can you tell us why your view of the CDC changed based on the agency's COVID-19 vaccine recommendation? | text |
| 221 | [fluvax] | Section Header: *Annual in uenza ( u) vaccination*<br><br>Do you normally get an in uenza ( u) vaccine each year? | yesno<br>1 Yes<br>0 No |
| 222 | [testreason] | Section Header: *Next, we would like to understand more about your experience(s) being tested for COVID-19.*<br><br>In the past when you've gotten tested for COVID, what were some of the reasons you got tested? Check all that apply. | checkbox<br>1 testreason___1 I had symptoms<br>2 testreason___2 I had contact with someone who tested positive<br>3 testreason___3 I had done something that was high risk<br>4 testreason___4 I was planning to do something that was high risk for myself or would put someone else at risk<br>5 testreason___5 I heard local COVID-19 cases were increasing<br>6 testreason___6 I was preparing for travel<br>7 testreason___7 I was required to as part of school/workplace requirements |

| | | | |
|---|---|---|---|
| | | | 8 \| testreason___8 \| Other |
| 223 | [nottested] | Public health guidance recommends testing if you have symptoms, have been exposed, have been in a high risk place, or plan to visit someone high risk. Have you ever NOT gotten tested for COVID-19 when [check all that apply] | checkbox<br>1 \| nottested___1 \| You had symptoms<br>2 \| nottested___2 \| You had contact with someone who tested positive<br>3 \| nottested___3 \| You did something that was high risk<br>4 \| nottested___4 \| You were planning to do something that was high risk for yourself or would put someone else at risk<br>5 \| nottested___5 \| You have always gotten tested when any of these things occurred<br>6 \| nottested___6 \| None of these things have ever occurred |
| 224 | [notest_reason]<br><br>Show the eld ONLY if:<br>[nottested(1)] = '1' or<br>[nottested(2)] = '1' or<br>[nottested(3)] = '1' or<br>[nottested(4)] = '1' | Why did you NOT get tested? | radio<br>1 \| I didn't feel sick<br>2 \| I was pretty sure I'd test negative<br>3 \| I was worried I'd test positive<br>4 \| I didn't want to do into isolation<br>5 \| I could quarantine and see if things got worse<br>6 \| I couldn't get tested (cost, access, test availability)<br>7 \| Other reasons |

| | | | |
|---|---|---|---|
| 225 | [test_required] | Were you ever tested as part of a mandatory testing program through your workplace ? | radio<br>1 \| Yes<br>2 \| No |

| | | | |
|---|---|---|---|
| | | | 3 Not sure<br>4 Rather not say |
| 226 | [prior_beh]<br><br>Show the eld ONLY if:<br>[test_required] = '1' | When you were tested as part of a mandatory program, did you change your behaviors prior to testing? | radio<br>1 Yes - I felt I could take more COVID-19 risks because I knew an infection would be detected quickly<br>2 Yes - I felt I needed to take fewer COVID-19 risks because I was worried I'd test positive<br>3 No - it didn't change my behavior at all |
| 227 | [test_planned] | Have you ever planned to take a COVID-19 test when you did not have symptoms, did not have contact with someone who tested positive, or need to ll a requirement for travel or healthcare? | radio<br>1 Yes<br>2 No<br>3 Not Sure<br>4 Rather not say |
| 228 | [beh_before]<br><br>Show the eld ONLY if:<br>[test_planned] = '1' | In the days BEFORE a planned test, did you typically change your behavior? | radio<br>1 Yes - I felt I could take more COVID-19 risks<br>2 Yes - I felt I needed to take fewer COVID-19 risks<br>3 No - it didn't change my behavior at all |
| 229 | [beh_after]<br><br>Show the eld ONLY if:<br>[test_planned] = '1' | In the days AFTER a COVID-19 test and a negative result, did you typically change your behavior? | radio<br>1 Yes - I felt I could take more COVID-19 risks<br>2 Yes - I felt I needed to take fewer COVID-19 risks<br>3 No - it didn't change my behavior at all |

| | | | |
|---|---|---|---|
| 230 | [mentalhealth] | Section Header: *Next, we'd like to understand more about your health and health behaviors. Remember that this data is not linked to your name and will be kept completely con dential.*<br><br>Has a doctor or other healthcare provider EVER told you that you have a mental health condition? | radio<br>1 Yes<br>2 No<br>3 I don't know |
| 231 | [mental_dx]<br><br>Show the eld ONLY if:<br>[mentalhealth] = '1' | If yes, have you been diagnosed with (choose all that apply): | checkbox<br>1 mental_dx___1 Depression<br>2 mental_dx___2 Anxiety<br>3 mental_dx___3 PTSD<br>4 mental_dx___4 Eating disorder<br>5 mental_dx___5 Substance use disorder<br>6 mental_dx___6 Another mental health condition<br>7 mental_dx___7 Prefer not to say |
| 232 | [smoke1] | Have you smoked at least 100 cigarettes in your entire life? | yesno<br>1 Yes<br>0 No |
| 233 | [smoke_2]<br><br>Show the eld ONLY if:<br>[smoke1] = '1' | Do you currently smoke cigarettes every day, some days, or not at all? | radio<br>1 Every day<br>2 Some days<br>3 Not at all |
| 234 | [smoke3]<br><br>Show the eld ONLY if:<br>[smoke_2] = '1' or [sm oke_2] = '2' | What type of tobacco products are you currently using? (Please check all that apply.) | checkbox<br>1 smoke3___1 Cigarettes<br>2 smoke3___2 Cigars<br>3 smoke3___3 Pipes<br>4 smoke3___4 Snu /Dip |

| | | | |
|---|---|---|---|
| | | | 5 smoke3___5 Chew<br>6 smoke3___6 Electronic Cigarettes<br>7 smoke3___7 Hookah/Water pipe<br>8 smoke3___8 Dissolvable tobacco (lozenge, strip, or sticks)<br>9 smoke3___9 Heat-not-burn tobacco (IQOS)<br>10 smoke3___10 Other |
| 235 | [audit1] | How often do you have a drink containing alcohol? | radio<br>1 Never<br>2 Monthly or less<br>3 2-4 times a month<br>4 2-3 times a week<br>5 4 or more times a week |
| 236 | [audit2]<br>Show the eld ONLY if:<br>[audit1] = '2' and [aud it1] = '3' and [audit1] = '4' and [audit1] = '5' | How many standard drinks containing alcohol do you have on a typical day? | radio<br>1 1 or 2<br>2 3 to 4<br>3 5 to 6<br>4 7 to 9<br>5 10 or more |
| 237 | [audit3]<br>Show the eld ONLY if:<br>[audit1] = '2' and [aud it1] = '3' and [audit1] = '4' and [audit1] = '5' | How often do you have six or more drinks on one occasion? | radio<br>1 Daily or almost daily<br>2 Weekly<br>3 Monthly<br>4 Less than monthly |

| | | | |
|---|---|---|---|
| | | | 5 Never |
| 238 | [drugs] | During the past 7 days, on how many days did you use any other drugs or prescription medication for non-medical reasons? | radio<br>0 0<br>1 1<br>2 2<br>3 3<br>4 4<br>5 5<br>6 6<br>7 7 |
| 239 | [coc]<br>Show the eld ONLY if: [drugs] = '1' or [drug s] = '2' or [drugs] = '3' or [drugs] = '4' or [dru gs] = '5' or [drugs] = '6' or [drugs] = '7' | Section Header: *Which of the drugs or prescription medications have you used for non-medical reasons in the past 7 days? (Check all that apply)*<br>Cocaine (coke, crack, etc.) | radio (Matrix)<br>1 Yes<br>2 No |
| 240 | [p_amp]<br>Show the eld ONLY if: [drugs] = '1' or [drug s] = '2' or [drugs] = '3' or [drugs] = '4' or [dru gs] = '5' or [drugs] = '6' or [drugs] = '7' | Prescribed amphetamine-type stimulants (Ritalin, Concerta, Dexedrine, Adderall, diet pills, etc.) | radio (Matrix)<br>1 Yes<br>2 No |
| 241 | [meth]<br>Show the eld ONLY if: [drugs] = '1' or [drug s] = '2' or [drugs] = '3' or [drugs] = '4' or [dru gs] = '5' or [drugs] = '6' or [drugs] = '7' | Methamphetamine (speed, crystal meth, ice, etc.) | radio (Matrix)<br>1 Yes<br>2 No |

| | | | |
|---|---|---|---|
| 242 | [inh]<br><br>Show the eld ONLY if:<br>[drugs] = '1' or [drug s] = '2' or [drugs] = '3' or [drugs] = '4' or [dru gs] = '5' or [drugs] = '6' or [drugs] = '7' | Inhalants (nitrous oxide, glue, gas, paint thinner, etc.) | radio (Matrix)<br>1 Yes<br>2 No |
| 243 | [sed]<br><br>Show the eld ONLY if:<br>[drugs] = '1' or [drug s] = '2' or [drugs] = '3' or [drugs] = '4' or [dru gs] = '5' or [drugs] = '6' or [drugs] = '7' | Sedatives or sleeping pills (Valium, Serepax, Ativan, Librium, Xanax, Rohypnol, GHB, etc.) | radio (Matrix)<br>1 Yes<br>2 No |
| 244 | [hal]<br><br>Show the eld ONLY if:<br>[drugs] = '1' or [drug s] = '2' or [drugs] = '3' or [drugs] = '4' or [dru gs] = '5' or [drugs] = '6' or [drugs] = '7' | Hallucinogens (LSD, acid, mushrooms, PCP, Special K, ecstasy, etc.) | radio (Matrix)<br>1 Yes<br>2 No |
| 245 | [s_op]<br><br>Show the eld ONLY if:<br>[drugs] = '1' or [drug s] = '2' or [drugs] = '3' or [drugs] = '4' or [dru gs] = '5' or [drugs] = '6' or [drugs] = '7' | Street Opioids (heroin, opium, etc.) | radio (Matrix)<br>1 Yes<br>2 No |
| 246 | [p_op]<br><br>Show the eld ONLY if:<br>[drugs] = '1' or [drug s] = '2' or [drugs] = '3' or [drugs] = '4' or [dru gs] = '5' or [drugs] = '6' or [drugs] = '7' | Prescribed opioids (fentanyl, oxycodone [OxyContin, Percocet], hydrocodone [Vicodin], methadone, buprenorphine, etc.) | radio (Matrix)<br>1 Yes<br>2 No |
| 247 | [mar]<br><br>Show the eld ONLY if:<br>[drugs] = '1' or [drug s] = '2' or [drugs] = '3' or | Marijuana (hashish, THC) | radio (Matrix)<br>1 Yes<br>2 No |

| | | | |
|---|---|---|---|
| | [drugs] = '4' or [dru gs] = '5' or [drugs] = '6' or [drugs] = '7' | | |
| 248 | [worry_current] | Section Header: *Next, we'd like to ask you about stress you may experience and how you may cope with that stress.*<br><br>What are some of your biggest sources of stress or worry right now? (This could include stressors or worries directly related to, or made worse by, the coronavirus outbreak. It can also include stressors completely unrelated to the coronavirus outbreak!) | notes |
| 249 | [cope] | People often have a lot of ways they deal with stress that are both healthy and not-sohealthy. What are some of the main ways you cope with your stress? | notes |
| 250 | [mspss_1] | Section Header: *We are interested in how you feel about the following statements. Read each statement carefully. Indicate how you feel about each statement.*<br><br>There is a special person who is around when I am in need | radio (Matrix)<br>1 Very strongly agree<br>2 Strongly agree<br>3 Agree<br>4 Neither agree nor disagree<br>5 Disagree<br>6 Strongly disagree<br>7 Very strongly disagree |
| 251 | [mspss_2] | There is a special person with whom I can share my joys and sorrows | radio (Matrix)<br>1 Very strongly agree<br>2 Strongly agree<br>3 Agree<br>4 Neither agree nor disagree<br>5 Disagree<br>6 Strongly disagree<br>7 Very strongly disagree |

| | | | |
|---|---|---|---|
| 252 | [mspss_3] | My family really tries to help me | radio (Matrix)<br>1 Very strongly agree<br>2 Strongly agree<br>3 Agree<br>4 Neither agree nor disagree<br>5 Disagree<br>6 Strongly disagree<br>7 Very strongly disagree |
| 253 | [mspss_4] | I get the emotional help and support I need from my family | radio (Matrix)<br>1 Very strongly agree<br>2 Strongly agree<br>3 Agree<br>4 Neither agree nor disagree<br>5 Disagree<br>6 Strongly disagree<br>7 Very strongly disagree |
| 254 | [mspss_5] | I have a special person who is a real source of comfort to me | radio (Matrix)<br>1 Very strongly agree<br>2 Strongly agree<br>3 Agree<br>4 Neither agree nor disagree<br>5 Disagree<br>6 Strongly disagree<br>7 Very strongly disagree |
| 255 | [mspss_6] | My friends really try to help me | radio (Matrix)<br>1 Very strongly agree |

| | | | |
|---|---|---|---|
| | | | 2 Strongly agree<br>3 Agree<br>4 Neither agree nor disagree<br>5 Disagree<br>6 Strongly disagree<br>7 Very strongly disagree |
| 256 | [mspss_7] | I can count on my friend when things go wrong | radio (Matrix)<br>1 Very strongly agree<br>2 Strongly agree<br>3 Agree<br>4 Neither agree nor disagree<br>5 Disagree<br>6 Strongly disagree<br>7 Very strongly disagree |
| 257 | [mspss_8] | I can talk about my problems with my family | radio (Matrix)<br>1 Very strongly agree<br>2 Strongly agree<br>3 Agree<br>4 Neither agree nor disagree<br>5 Disagree<br>6 Strongly disagree<br>7 Very strongly disagree |
| 258 | [mspss_9] | I have friends with whom I can share my joys and sorrows | radio (Matrix)<br>1 Very strongly agree<br>2 Strongly agree |

| | | | |
|---|---|---|---|
| | | | 3 Agree<br>4 Neither agree nor disagree<br>5 Disagree<br>6 Strongly disagree<br>7 Very strongly disagree |
| 259 | [mspss_10] | There is a special person in my life who cares about my feelings | radio (Matrix)<br>1 Very strongly agree<br>2 Strongly agree<br>3 Agree<br>4 Neither agree nor disagree<br>5 Disagree<br>6 Strongly disagree<br>7 Very strongly disagree |
| 260 | [mspss_11] | My family is willing to help me make decisions | radio (Matrix)<br>1 Very strongly agree<br>2 Strongly agree<br>3 Agree<br>4 Neither agree nor disagree<br>5 Disagree<br>6 Strongly disagree<br>7 Very strongly disagree |
| 261 | [mspss_12] | I can talk about my problems with my friends | radio (Matrix)<br>1 Very strongly agree<br>2 Strongly agree<br>3 Agree<br>4 Neither agree nor disagree |

| | | | |
|---|---|---|---|
| | | | 5 Disagree<br>6 Strongly disagree<br>7 Very strongly disagree |
| 262 | [abuse_pastyr] | Section Header: *This next section will ask about experiences you may have had with a current or former partner.*<br><br>In the past year, have you been emotionally abused by a current or former partner? | radio<br>1 Yes<br>2 No |
| 263 | [abuse_who]<br><br>Show the eld ONLY if:<br>[abuse_pastyr] = '1' | If yes: Who? | radio<br>1 Current partner<br>2 Former partner |
| 264 | [physical_pastyr] | In the past year, have you been hit, slapped, kicked or otherwise physically hurt by a current or former partner? | radio<br>1 Yes<br>2 No |
| 265 | [physical_who]<br><br>Show the eld ONLY if:<br>[physical_pastyr] = '1' | If yes, Who? | radio<br>1 Current partner<br>2 Former partner |
| 266 | [sexual_pastyr] | In the past year, has a current or former partner forced you to have sexual activities? | radio<br>1 Yes<br>2 No |
| 267 | [sexual_who]<br><br>Show the eld ONLY if:<br>[sexual_pastyr] = '1' | If yes: Who? | radio<br>1 Current partner<br>2 Former partner |
| 268 | [afraid_pastyr] | | radio |

| | | | |
|---|---|---|---|
| | | In the past year, have you been afraid of a current or former partner? | 1 Yes<br>2 No |
| 269 | [afraid_who]<br><br>Show the eld ONLY if:<br>[afraid_pastyr] = '1' | If yes, Who? | radio<br>1 Current partner<br>2 Former partner |
| 270 | [sexual_other_2]<br><br>Show the eld ONLY if:<br>[afraid_pastyr] = '1' or<br>[abuse_pastyr] = '1' o r<br>[physical_pastyr] =<br>'1' or [sexual_pastyr]<br>= '1' | Please tell us about it: | notes |
| 271 | [socialsupport_text] | What are things like for you at home in terms of relationships, support, social dynamics? How has coronavirus changed or impacted your relationships, social life, and social support? | text |
| 272 | [proqol1] | Section Header: *When you help people you have direct contact with their lives. As you may have found, your compassion for those you help can a ect you in positive and negative ways. Below are some questions about your experiences, both positive and negative, as a health care worker. Consider each of the following questions about you and your current work situation. Select the number that honestly re ects how frequently you experienced these things in the last 30 days.*<br><br>I am happy. | radio (Matrix)<br>1 Never<br>2 Rarely<br>3 Sometimes<br>4 Often<br>5 Very Often |
| 273 | [proqol2] | I am preoccupied with more than one person I cared for at work. | radio (Matrix)<br>1 Never<br>2 Rarely<br>3 Sometimes<br>4 Often<br>5 Very Often |
| 274 | [proqol3] | I get satisfaction from being able to help people. | radio (Matrix)<br>1 Never |

| | | | |
|---|---|---|---|
| | | | 2 Rarely<br>3 Sometimes<br>4 Often<br>5 Very Often |
| 275 | [proqol4] | I feel connected to others. | radio (Matrix)<br>1 Never<br>2 Rarely<br>3 Sometimes<br>4 Often<br>5 Very Often |
| 276 | [proqol5] | I jump or am startled by unexpected sounds. | radio (Matrix)<br>1 Never<br>2 Rarely<br>3 Sometimes<br>4 Often<br>5 Very Often |
| 277 | [proqol6] | I feel invigorated after working with those I care for. | radio (Matrix)<br>1 Never<br>2 Rarely<br>3 Sometimes<br>4 Often<br>5 Very Often |
| 278 | [proqol7] | I nd it di cult to separate my personal life from my life as a health care worker. | radio (Matrix)<br>1 Never<br>2 Rarely<br>3 Sometimes<br>4 Often |

| | | | |
|---|---|---|---|
| | | | 5 Very Often |
| 279 | [proqol8] | I am not as productive at work because I am losing sleep over traumatic experiences of a person I cared for at work. | radio (Matrix)<br>1 Never<br>2 Rarely<br>3 Sometimes<br>4 Often<br>5 Very Often |
| 280 | [proqol9] | I think that I might have been a ected by the traumatic stress of those I care for. | radio (Matrix)<br>1 Never<br>2 Rarely<br>3 Sometimes<br>4 Often<br>5 Very Often |
| 281 | [proqol10] | I feel trapped by my job as a health care worker. | radio (Matrix)<br>1 Never<br>2 Rarely<br>3 Sometimes<br>4 Often<br>5 Very Often |
| 282 | [proqol11] | Because of my work, I have felt "on edge" about various things. | radio (Matrix)<br>1 Never<br>2 Rarely<br>3 Sometimes<br>4 Often<br>5 Very Often |

| | | | |
|---|---|---|---|
| 283 | [proqol12] | I like my work in health care. | radio (Matrix)<br>1 Never<br>2 Rarely<br>3 Sometimes<br>4 Often<br>5 Very Often |
| 284 | [proqol13] | I feel depressed because of the traumatic experiences of the people I care for. | radio (Matrix)<br>1 Never<br>2 Rarely<br>3 Sometimes<br>4 Often<br>5 Very Often |
| 285 | [proqol14] | I feel as though I am experiencing the trauma of someone I have cared for. | radio (Matrix)<br>1 Never<br>2 Rarely<br>3 Sometimes<br>4 Often<br>5 Very Often |
| 286 | [proqol15] | I have beliefs that sustain me. | radio (Matrix)<br>1 Never<br>2 Rarely<br>3 Sometimes<br>4 Often<br>5 Very Often |
| 287 | [proqol16] | | radio (Matrix) |

| | | | |
|---|---|---|---|
| | | I am pleased with how I am able to keep up with health care techniques and protocols. | 1 Never<br>2 Rarely<br>3 Sometimes<br>4 Often<br>5 Very Often |
| 288 | [proqol17] | I am the person I always wanted to be. | radio (Matrix)<br>1 Never<br>2 Rarely<br>3 Sometimes<br>4 Often<br>5 Very Often |
| 289 | [proqol18] | My work makes me feel satis ed. | radio (Matrix)<br>1 Never<br>2 Rarely<br>3 Sometimes<br>4 Often<br>5 Very Often |
| 290 | [proqol19] | I feel worn out because of my work. | radio (Matrix)<br>1 Never<br>2 Rarely<br>3 Sometimes<br>4 Often<br>5 Very Often |
| 291 | [proqol20] | I have happy thoughts and feelings about those I care for and how I could help them. | radio (Matrix)<br>1 Never |

| | | | |
|---|---|---|---|
| | | | 2 Rarely<br>3 Sometimes<br>4 Often<br>5 Very Often |
| 292 | [proqol21] | I feel overwhelmed because my work load seems endless. | radio (Matrix)<br>1 Never<br>2 Rarely<br>3 Sometimes<br>4 Often<br>5 Very Often |
| 293 | [proqol22] | I believe I can make a di erence through my work. | radio (Matrix)<br>1 Never<br>2 Rarely<br>3 Sometimes<br>4 Often<br>5 Very Often |
| 294 | [proqol23] | I avoid certain activities or situations because they remind me of frightening experiences of the people I care for. | radio (Matrix)<br>1 Never<br>2 Rarely<br>3 Sometimes<br>4 Often<br>5 Very Often |
| 295 | [proqol24] | I am proud of what I can do at work. | radio (Matrix)<br>1 Never<br>2 Rarely<br>3 Sometimes |

| | | | |
|---|---|---|---|
| | | | 4 Often<br>5 Very Often |
| 296 | [proqol25] | As a result of my work, I have intrusive, frightening thoughts. | radio (Matrix)<br>1 Never<br>2 Rarely<br>3 Sometimes<br>4 Often<br>5 Very Often |
| 297 | [proqol26] | I feel "bogged down" by the system. | radio (Matrix)<br>1 Never<br>2 Rarely<br>3 Sometimes<br>4 Often<br>5 Very Often |
| 298 | [proqol27] | I have thoughts that I am a "success" as a health care worker. | radio (Matrix)<br>1 Never<br>2 Rarely<br>3 Sometimes<br>4 Often<br>5 Very Often |
| 299 | [proqol28] | I can't recall import parts of my work with patients. | radio (Matrix)<br>1 Never<br>2 Rarely<br>3 Sometimes<br>4 Often |

| | | | |
|---|---|---|---|
| | | | 5 Very Often |
| 300 | [proqol29] | I am a very caring person. | radio (Matrix)<br>1 Never<br>2 Rarely<br>3 Sometimes<br>4 Often<br>5 Very Often |
| 301 | [proqol30] | I am happy that I chose to do this work. | radio (Matrix)<br>1 Never<br>2 Rarely<br>3 Sometimes<br>4 Often<br>5 Very Often |
| 302 | [abuse_work] | Section Header: *For this set of questions we want to know about things that may have happened to you at work. The person who did these things may be a co-worker, patient, patient family member, or someone else entirely - but they happened in your workplace.*<br>In the past year, have you been emotionally abused by someone at work? | radio<br>1 Yes<br>2 No |
| 303 | [abusew_who]<br>Show the eld ONLY if:<br>[abuse_work] = '1' | If yes: Who? | radio<br>1 Boss/Supervisor<br>2 Co-worker<br>3 Patient<br>4 Patient family member |
| 304 | [abuse_work_other]<br>Show the eld ONLY if:<br>[abuse_work] = '1' | Please tell us about it: | notes |
| 305 | [physical_work] | | radio |

| | | | |
|---|---|---|---|
| | | In the past year, have you been hit, slapped, kicked or otherwise physically hurt by someone at work? | 1 Yes<br>2 No |
| 306 | [physicalw_who]<br><br>Show the eld ONLY if:<br>[physical_work] = '1' | If yes: Who? | radio<br>1 Boss/Supervisor<br>2 Co-worker<br>3 Patient<br>4 Patient family member |
| 307 | [physical_work_other]<br><br>Show the eld ONLY if:<br>[physical_work] = '1' | Please tell us about it: | notes |
| 308 | [sexual_work] | In the past year, has someone at work forced you to have sexual activities? | radio<br>1 Yes<br>2 No |
| 309 | [sexual_work_who]<br><br>Show the eld ONLY if:<br>[sexual_work] = '1' | If yes: Who? | radio<br>1 Boss/Supervisor<br>2 Co-worker<br>3 Patient<br>4 Patient family member |
| 310 | [sexual_work_other]<br><br>Show the eld ONLY if:<br>[sexual_work] = '1' | Please tell us about it: | notes |
| 311 | [afraid_work] | In the past year, have you been afraid of someone at work? | radio<br>1 Yes<br>2 No |
| 312 | [afraidw_who]<br><br>Show the eld ONLY if: | If yes: Who? | radio<br>1 Boss/Supervisor |

| | | | |
|---|---|---|---|
| | [afraid_work] = '1' | | 2 Co-worker<br>3 Patient<br>4 Patient family member |
| 313 | [afraid_work_other]<br><br>Show the eld ONLY if:<br>[afraid_work] = '1' | Please tell us about it: | notes |
| 314 | [work_life] | What are things like for you at work in terms of support, group dynamics, sta ng, con ict, equipment? How has coronavirus changed or impacted your work life? | notes |
| 315 | [covid_routine] | Section Header: *Next, we would like to understand more about how the COVID-19 pandemic may have impacted your life. For each of the following, rate how much the Coronavirus pandemic has changed your life.*<br><br>Routines: | radio<br>0 No change.<br>1 Mild. Change in only one area (e.g. work, education, social life, hobbies, religious activities).<br>2 Moderate. Change in two areas (e.g. work, education, social life, hobbies, religious activities).<br>3 Severe. Change in three or more areas (e.g. work, education, social life, hobbies, religious activities). |
| 316 | [covid_income] | Family Income/Employment: | radio<br>0 No change.<br>1 Mild. Small change; able to meet all needs and pay bills.<br>2 Moderate. Having to make cuts but able to meet basic needs and pay bills.<br>3 Severe. Unable to meet basic needs and/or pay bills. |
| 317 | [covid_food] | Food Access: | radio<br>0 No change. |

| | | | |
|---|---|---|---|
| | | | 1 Mild. Enough food but di culty getting to stores and/or nding needed items.<br>2 Moderate. Occasionally without enough food and/or good quality (e.g., healthy) foods.<br>3 Severe. Frequently without enough food and/or good quality (e.g., healthy) foods. |
| 318 | [covid_care] | Medical health care access: | radio<br>0 No change.<br>1 Mild. Appointments moved to telehealth.<br>2 Moderate. Delays or cancellations in appointments and/or delays in getting prescriptions; changes have minimal impact on health.<br>3 Severe. Unable to access needed care resulting in moderate to severe impact on health. |
| 319 | [covid_trt] | Mental health treatment access: | radio<br>0 No change.<br>1 Mild. Appointments moved to telehealth.<br>2 Moderate. Delays or cancellations in appointments and/or delays in getting prescriptions; changes have minimal impact.<br>3 Severe. Unable to access needed care resulting in severe risk and/or signi cant impact. |
| 320 | [covid_social] | Access to extended family and non-family social supports: | radio<br>0 No change.<br>1 Mild. Continued visits with social distancing and/or regular phone calls and/or televideo or social media contacts. |

| | | | |
|---|---|---|---|
| | | | 2 Moderate. Loss of in person and remote contact with a few people, but not all supports.<br>3 Severe. Loss of in person and remote contact with all supports. |

| | | | |
|---|---|---|---|
| 321 | [covid_stress] | Experiences of stress related to coronavirus pandemic: | radio<br>0 None.<br>1 Mild. Occasional worries and/or minor stress-related symptoms (e.g., feel a little anxious, sad, and/or angry; mild/rare trouble sleeping).<br>2 Moderate. Frequent worries and/or moderate stress-related symptoms (e.g., feel moderately anxious, sad, and/or angry; severe/frequent trouble sleeping).<br>3 Severe. Persistent worries and/or severe stress-related symptoms (e.g., feel extremely |
| 322 | [covid_famstress] | Stress and discord in the family: | radio<br>0 None.<br>1 Mild. Family members occasionally short-tempered with one another; no physical violence.<br>2 Moderate. Family members frequently short-tempered with one another; and/or children in the home getting in physical ghts with one another.<br>3 Severe. Family members frequently short-tempered with one another and adults in the home throwing things at one another, and/or knocking over furniture, and/or hitting and/or harming one another. |
| 323 | [covid_dx] | Personal diagnosis of coronavirus. | radio |

| | | | |
|---|---|---|---|
| | | | 0 None.<br>1 Mild. Symptoms e ectively managed at home.<br>2 Moderate. Symptoms severe and required brief hospitalization.<br>3 Severe. Symptoms severe and required ventilation. |
| 324 | [covid_family] | Number of immediate family members diagnosed with coronavirus: | text |
| 325 | [covid_severe]<br><br>Show the eld ONLY if:<br>[covid_family] > 0 or 'zero' | Rate the symptoms of the person who was most sick: | radio<br>1 Mild. Symptoms e ectively managed at home.<br>2 Moderate. Symptoms severe and required brief hospitalization.<br>3 Severe. Symptoms severe and required ventilation.<br>4 Immediate family member died from coronavirus. |
| 326 | [covid_extended] | Number of extended family member(s) and/or close friends diagnosed with coronavirus: | text |
| 327 | [covid_severe2]<br><br>Show the eld ONLY if:<br>[covid_extended] > 0 or 'zero' | Rate the symptoms of the person who was most sick: | radio<br>1 Mild. Symptoms e ectively managed at home.<br>2 Moderate. Symptoms severe and required brief hospitalization.<br>3 Severe. Symptoms severe and required ventilation.<br>4 Extended family member and/or close friend died of coronavirus. |
| 328 | [covid_additional] | Is there anything else you want to share with us about your life or experience during the COVID-19 pandemic? | notes |
| 329 | [healthcare_worker_survey_english_penn_state_complete] | Section Header: *Form Status*<br><br>Complete? | dropdown<br>0 Incomplete |

| | | | |
|---|---|---|---|
| | | | 1 Unveri ed<br>2 Complete |
